# N protein from lambdoid phages transforms NusA into an antiterminator by modulating NusA-RNA polymerase flap domain interactions

**DOI:** 10.1093/nar/gkv479

**Published:** 2015-05-18

**Authors:** Saurabh Mishra, Ranjan Sen

**Affiliations:** 1 Laboratory of Transcription, Center for DNA Fingerprinting and Diagnostics, Tuljaguda Complex, 4-1-714 Mozamjahi Road, Nampally, Hyderabad 500 001, India; 2 Graduate Studies, Manipal University, India

## Abstract

Interaction of the lambdoid phage N protein with the bacterial transcription elongation factor NusA is the key component in the process of transcription antitermination. A convex surface of *E. coli* NusA-NTD, located opposite to its RNA polymerase-binding domain (the β-flap domain), directly interacts with N in the antitermination complex. We hypothesized that this N-NusA interaction induces allosteric effects on the NusA-RNAP interaction leading to transformation of NusA into a facilitator of the antitermination process. Here we showed that mutations in β-flap domain specifically defective for N antitermination exhibited altered NusA-nascent RNA interaction and have widened RNA exit channel indicating an intricate role of flap domain in the antitermination. The presence of N reoriented the RNAP binding surface of NusA-NTD, which changed its interaction pattern with the flap domain. These changes caused significant spatial rearrangement of the β-flap as well as the β′ dock domains to form a more constricted RNA exit channel in the N-modified elongation complex (EC), which might play key role in converting NusA into a facilitator of the N antitermination. We propose that in addition to affecting the RNA exit channel and the active center of the EC, β-flap domain rearrangement is also a mechanistic component in the N antitermination process.

## INTRODUCTION

NusA is a highly conserved bacterial transcription elongation factor that is involved in various steps of the transcription process (1). It improves intrinsic termination at weak terminators by facilitating the hairpin formation (2,3) and enhances pausing during transcription elongation especially at the RNA hairpin-dependent pauses (4–6). It gets converted into an antiterminator in the presence of bacteriophage antiterminator proteins like N and Q (7). Recently, it has been shown to be involved in DNA repair process too (8). *E. coli* NusA is a ∼55 kD protein, having multiple functional domains; N-terminal RNAP-binding domain (NTD), three RNA binding domains (S1, KH1 and KH2) and two C-terminal acidic repeats (AR1 and AR2) ((9,10); Supplementary Figure S1 A-C). A concave surface of the NTD interacts with the flap domain of the β-subunit of the *E. coli* RNAP (11,12), whereas the AR2 binds to the C-terminal domain (α-CTD) of the α-subunit of the RNAP (13).

Antiterminator proteins, like N and Q, make the transcription elongation complex (EC) resistant to terminators. This phenomenon is called transcription antitermination ((7,14); Supplementary Figure S1D). N is a small basic protein that binds to a specific stem-loop structure (box B of nut site; Supplementary Figure S1E) in the mRNA through its N-terminal arginine rich motif (ARM; (15,16); Supplementary Figure S1) and interacts with the RNAP through its C-terminus (17,18). N requires several host factors, called Nus factors, for the processive antitermination ((14), Supplementary Figure S1F). N and these Nus factors assemble on the ‘nut’ (N utilization) site of the mRNA, where N and NusA make specific interactions ((7); Supplementary Figure S1E). In case of Q, the Q-NusA interaction occurs on the surface of the EC that facilitates the action of the former (7).

Among the Nus factors, NusA-N interaction is most important for the processive antitermination function of N, and this interaction transforms NusA into an antiterminator from its natural role as a facilitator of transcription termination. Recently, we have identified that a convex surface of NusA-NTD, located opposite to its RNA polymerase-binding domain, interacts directly with N when the antitermination complex is formed on the surface of the EC (19). We proposed that this N-NusA-NTD interaction enables N to exert allosteric effects on the EC via NusA ((19), Supplementary Figure S2). The molecular nature of N induced alteration in NusA-RNAP interaction is not known, understanding of which is essential to decipher the antitermination mechanism.

In this report, using genetics and biochemical probing of the *E. coli* transcription machinery, we have conclusively shown that N-NusA NTD interaction on the surface of the EC modulates the NusA- RNAP β−flap interaction by spatial reorientation of the flap-tip region located in the RNA exit channel. These interactions constrict the RNA-exit channel of the EC, which most likely hinders the formation of terminator hairpin in the exit channel. And hence, explains why N affects the terminator hairpin formation that was shown earlier (3,20,21).

## MATERIALS AND METHODS

### Synthetic defect assays

To test whether the combination of *rpoB-*G1045D (*E. coli* RNAP β-subunit) and *nusA*–V8A mutants induces synthetic defects in N-mediated *in vivo* antitermination, pHYD3011 plasmid expressing either WT or V8A NusA was transformed into the strains, RS1452 (having *rpoB-*G1045D and *P_lac_-*H-19B *nutR-T_R′_-lacZYA* reporter cassette) and RS1018 (having WT *rpoB* and *P_lac_-*H-19B *nutR-T_R′_-lacZYA* reporter). Following that chromosomal WT *nusA* of these *E. coli* strains was deleted by P1 transduction *(nusA::Cam^R^*). β-galactosidase assays were performed following the standard procedures using microtiter plates (21).

### DNA templates used in various *in vitro* assays

Linear DNA templates for different *in vitro* assays were made by PCR amplifications from the plasmids, pRS604 (T7A1-*λnutR-lacO*; oligo pairs RS83/RS333 for Template I and RS83/RS994 for Template II), pRS106 (pT7A1-*trpt’*-*lacO*; oligo pair RS83/177 for Template III) and pRS22 (pT7A1-H 19B *nutR-T_R′_-T1-T2*; oligo pairs, RS58/RK1, Template IV). The *lacO* sequence is present in the downstream oligos RS177, RS333 and RS994. The *ops* pause sequence from *E. coli* (22) was incorporated downstream to the H-19B *nutR* site (T7A1- H 19B *nutR-ops-lacO*, Template V) by overlapping PCR amplification using the primers RS83, RS267 and RS276. The *his* pause sequence was incorporated after the *nutR* site (T7A1-H-19B *nutR-his pause-lacO*, Template VI) also by overlapping PCR method using the primers, RS83, RS263, RS264, RS265 and RS275. In both these cases, pRS22 was used for amplification, and the oligos RS275 and RS276 contained the *lacO* sequence so that both these templates have a *lac* operator sequence at their downstream edge. The template used in RNase H cleavage assays, Template VII, was amplified from pRS22 using RS83/RS404 oligo pairs. Template VIII, used in the Fe-BABE cleavage assays, was made from pRS385 using oligo pairs RS83/RS2. RS404 has the *lac* operator sequence. This operator sequence is cloned downstream of the H-19B *nutR* site in pRS385 (23). In all these aforementioned templates, stalled ECs (RB, road-blocked complex) were formed at the operator site in the presence of Lac repressor. In some cases, pRS25 (21) was used for making the T7A1-H 19B *nutR-T_R′_* template using the oligo pair RS58/RS2 by PCR-amplification. Transcription was initiated from the T7A1 promoter in all these templates. When required, the templates were immobilized on the streptavidin beads via a 5′-biotin group using the biotinylated primer RS83.

### *In vitro* transcription assays

For the *in vitro* transcription on the T7A1-*nutR-T_R′_-T1-T2* template, reactions were carried out in T-Glu buffer [20 mM Tris-glutamate (pH 8.0), 10 mM magnesium glutamate, 50 mM potassium glutamate, 1 mM DTT and 100 μg/ml BSA) at 32°C. The reactions were initiated with 175 μM ApU, 5 μM GTP, 5 μM ATP, 2.5 μM CTP and [γ-^32^P]CTP (∼3000Ci/mmol) to make a 23-mer EC (EC_23_). Then it was chased with 250 μM each of all the NTPs in the presence of 300 nM of NusA and 200 nM NusG. The reactions were stopped by phenol extraction followed by ethanol precipitation. Samples were loaded onto a 6% sequencing gel and analyzed using FLA 9000 phosphorimager (Fuji).

### Formation of the stalled EC (RB) for pausing assays, NusA-dissociation, RNaseH and Fe-BABE cleavage assays

To form the RBs for different assays, we have used different DNA templates (Template I, II, etc.) immobilized to the streptavidin-coated magnetic beads via a biotin group at its 5′ end. These templates also contain a 22-bp lac operator sequence at its downstream end. On these templates, EC_23_ was formed at first in the same way as described before, except that the reactions were performed in T-Cl buffer [50 mM Tris.HCl (pH 8.0), 100 mM KCl, 10 mM MgCl_2_, 1 mM DTT and 100 μg/ml BSA] and in the presence of 100 nM Lac repressor. EC_23_ was then chased with 250 μM of NTPs for 2 min to form the RB. The RB was then washed to remove excess NTPs and used in different assays. The transcription reactions were carried out at 37°C.

### NusA dissociation assays

**Figure 1.**
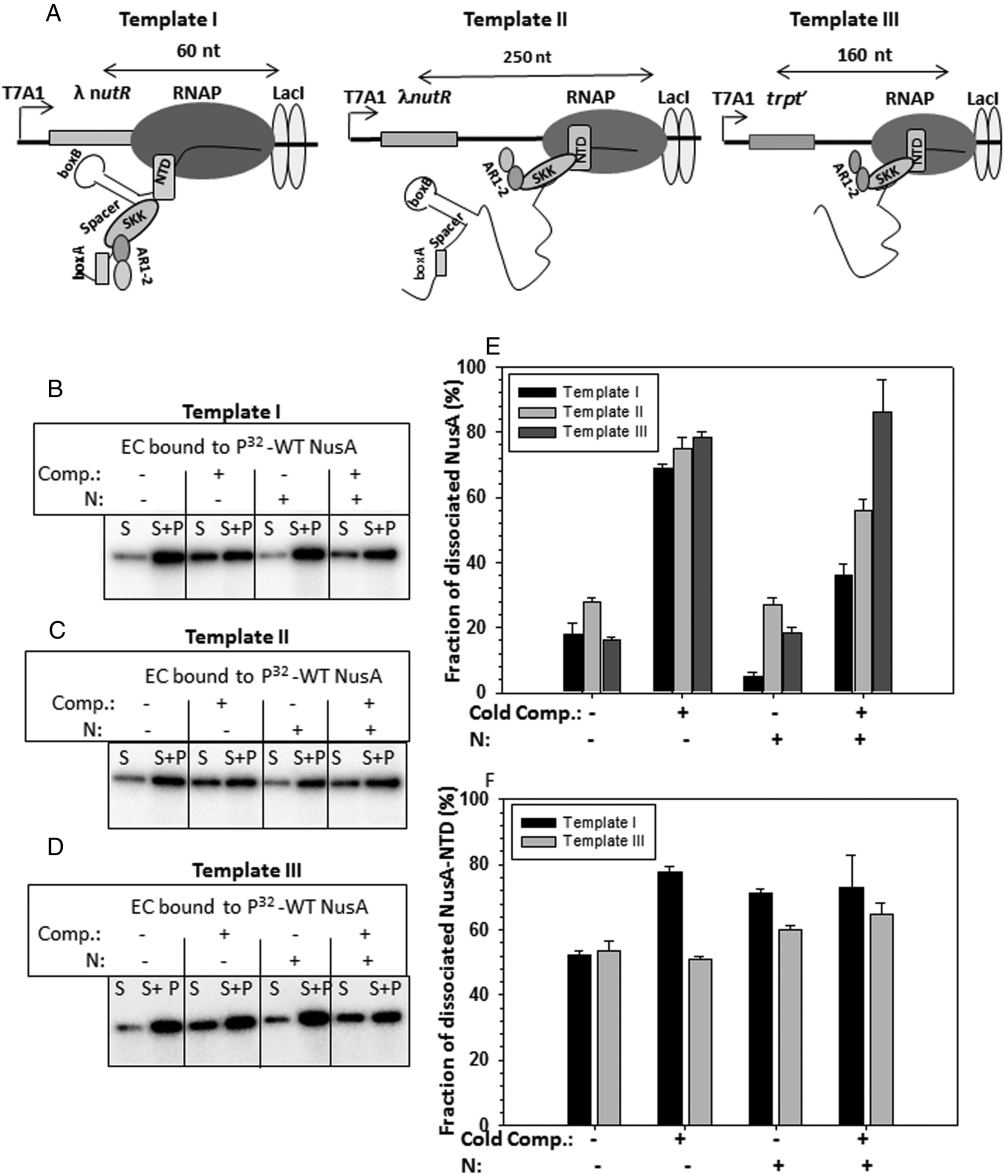
NusA cold competition assays on stalled ECs. (**A**) Schematics of immobilized DNA templates on which the stalled ECs were constructed using lac repressor as a road block are described. Immobilization was performed via 5′-biotin linkages to streptavidin-coated magnetic beads. Different domains of NusA are indicated. Distances at which the ECs were stalled are also mentioned. Different elements of the *nut* sites in Template I and II are described. Transcription was initiated from T7A1 promoter. Distributions of radiolabeled NusA in supernatant (S; half of the supernatant) and pellet (P, rest of the S + pellet) fractions measuring the amount of dissociated NusA from the ECs made on the Templates I (**B**), II (**C**) and III (**D**). ECs were formed both in the presence and absence of λN. Bar diagrams showing amounts of either full-length NusA (**E**) or NusA-NTD (**F**) dissociated under different conditions both in the presence and absence of the cold competitor (cold comp.). Bar diagrams of **(E)** correspond to the gel pictures shown in (B)–(D), whereas those in (F) were calculated from the gels shown in Supplementary Figure S3A and B. Error bars were obtained from three independent measurements.

In these experiments, EC_23_ was chased in the presence of 10 nM of ^32^P-labeled WT NusA for 2 min to form the RBs on the immobilized Templates I, II and III. Excess NTPs were then removed by washing the beads, which was followed by the addition of 100 nM of unlabeled (cold) WT NusA with or without N as competitors. After 5 min, 15 μl of samples were removed and separated into supernatant (S) and pellet (P) fractions by keeping the tubes on magnetic stands. Half of the supernatant (S) and rest of the sample (rest of the supernatant plus the whole pellet fraction, denoted as S+P in the formula and as P in Figure 1) were mixed with equal volume of sodium dodecyl sulphate (SDS) loading buffer and loaded onto 10% sodium dodecylsulphate-polyacrylamide gel electrophoresis (SDS-PAGE). Amounts of NusA in different fractions were measured by scanning the gels in a Fuji Phosphoimager. Fractions of NusA dissociated, (2S) / [(S) + (S+P)], were plotted against different competitors as vertical bars.

To compare binding affinity of WT NusA for the stalled ECs made of WT and G1045D RNAP, fractions of dissociated radio-labeled WT NusA were plotted against increasing concentrations of cold NusA as competitor following the same experimental procedures as above. The curves were fitted to a hyperbolic equation of the form: *y* = (*ax*) / (*b* + *x*), where, *x* = [Competitor], *y* = fraction of dissociated NusA, *b* = concentration of the competitor at the half-maxima and *a* = maxima.

### Pausing assays

For pause assays, RBs made of WT or G1045D RNAP were at first formed on the Template V or VI at the respective pause sites in the same way as described above. Concentrations of DNA and RNAP were 10 nM and 50 nM, respectively. The RBs were then washed thoroughly to remove the excess NTPs, following which they were chased out of the stalled position in the presence of 100 μM each of UTP, CTP, ATP, 10 μM GTP and 1 mM IPTG. When required, 300 nM of NusA was added to the chase. Aliquots were removed during the specified time points (0, 30, 45, 60, 90, 120, 180, 240 and 300″ for the *his* pause and 0, 30, 45, 60, 90, 120, 180, 240″ for the *ops* pause) and mixed with formamide loading dye (Ambion). RNA products were separated on 8% sequencing gel. Fraction of paused complex was calculated as: (Intensity of the paused RNA) / (Total intensities of pause RNA + other higher–sized products). Plots obtained from plotting the fraction of paused products against the time were fitted to an exponential decay curve of the form: *y* = *y*_o_ + *A*.exp^−λt^, where *A* = amplitude, λ = rate constant, *y* = fraction of paused complex and *y*_o_ value corresponds to the plateau of a curve representing unchased complexes. The pause half-lives (*t*_1/2_) were measured from *t*_1/2_ = ln2/λ.

### RNase H cleavage assays

RNase H assays were performed with the RBs made on Template VII. Cleavage positions were defined by designing series of antisense DNA oligos, RS549, RS550, RS551 and RS1106, 5′-end of which correspond to the -10, -11, -12 and -14 positions of the nascent RNA, respectively (see Figure 4). RBs were formed in the same way as described above. The RBs were re-suspended in the T-Cl. buffer with or without 300 nM of WT NusA and 300 nM of H-19B N for 5 min. Following that RNase H cleavage was initiated by adding 2.5 μM of each of the antisense oligos and 0.25 U of RNase H, and incubation was continued at 37°C for different time points. Reaction volume was 10 μl. Reactions were stopped by phenol extraction, samples were mixed with equal volume of formamide loading dye and loaded onto 8% sequencing gel.

**Figure 2.**
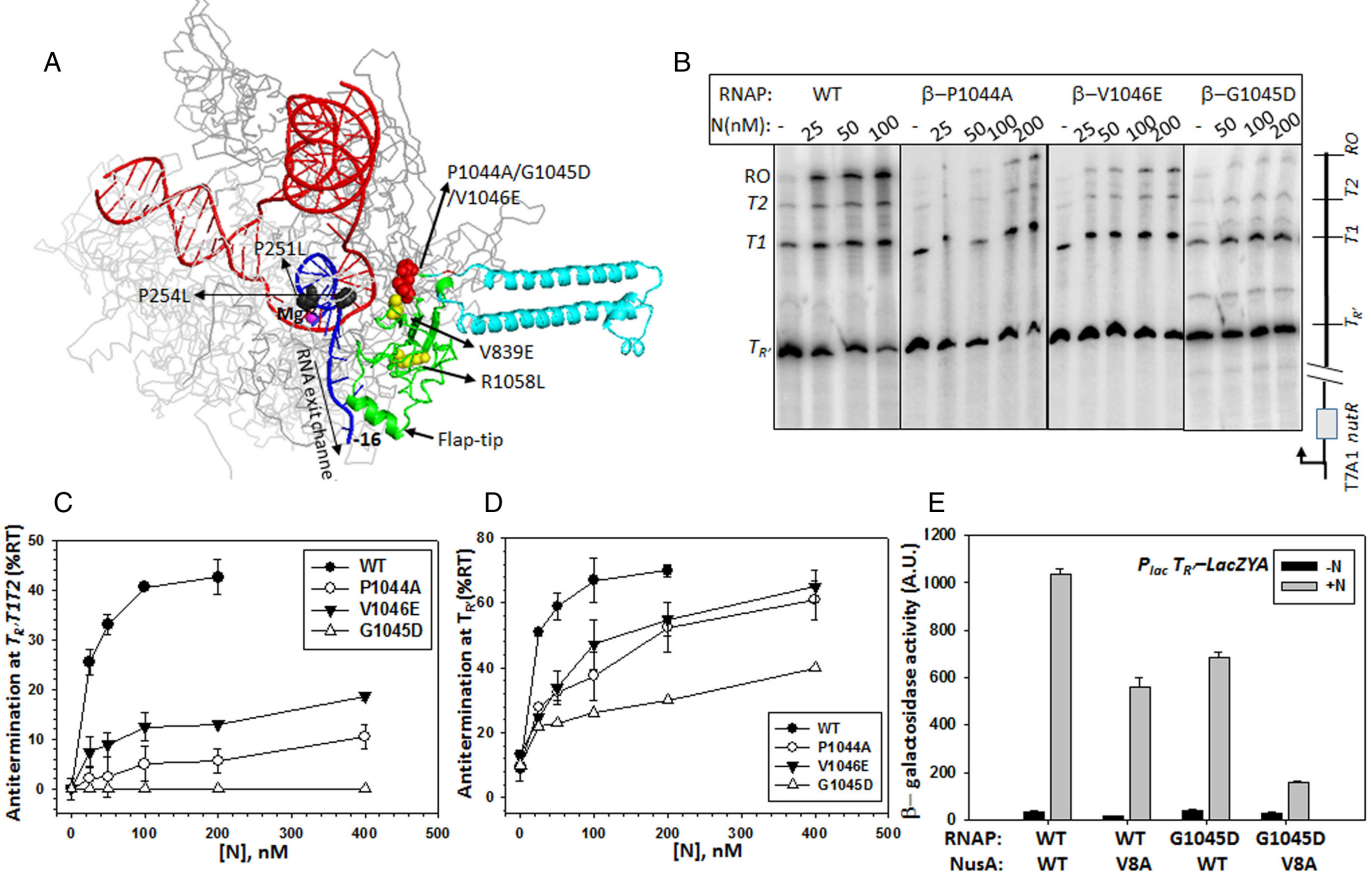
Involvement of β-flap domain in N antitermination. (**A**) Structural model of the EC developed through a hybrid approach combining X-ray crystallography, *ab initio* structural prediction, homology modeling and single-particle cryo-electron microscopy (29). Relevant portions of the structure have been highlighted. The flap domain is shown in green. DNA is shown as red double helix, whereas the nascent RNA (up to -16th residue) is in blue. The RNA is shown to be coming out of the exit channel. Different RNAP mutants are indicated in colored spheres. P251L and P254L are in β′-subunit. Others are in β-subunit. Active site Mg(II) is in violet sphere. Rest of the RNAP is shown as light gray in the background. An insert in the flap domain is shown in sky blue. (**B**) Autoradiogram showing the transcription read-through activities of the WT and the flap mutants at the triple terminators, *T_R′_, T*_1_ and *T*_2_. Concentrations of N in each lane are indicated. The schematic of the DNA template is shown next to the gel. H-19B *nutR* site is cloned upstream of the terminator cassette. Transcription was initiated from T7A1 promoter. NusG was added to the reactions to stimulate the H-19B N *in vitro* activity (21). RO denotes the amounts of transcript reached at the end of the template. Amounts of read-through (%RT) products at the end of the triple terminators (**C**) and after the *T_R′_* terminator (**D**) are plotted against increasing concentrations of N. %RT was calculated as for (C), [RO]/[*T_R′_* + *T*_1_ + *T*_2_ + RO] and for (D), [*T*_1_ + *T*_2_ + RO]/[*T_R′_* + *T*_1_ + *T*_2_ + RO]. (**E**) Bar diagram showing the β−galactosidase activities from the reporter strain expressing indicated combinations of different RNAP and NusA derivatives. *LacZYA* reporter is cloned downstream of the *H-19B nutR-T_R′_* sequence. Activities were measured both in the presence and the absence of N protein. Errors were calculated from the activities obtained from five to six independent colonies.

**Figure 3.**
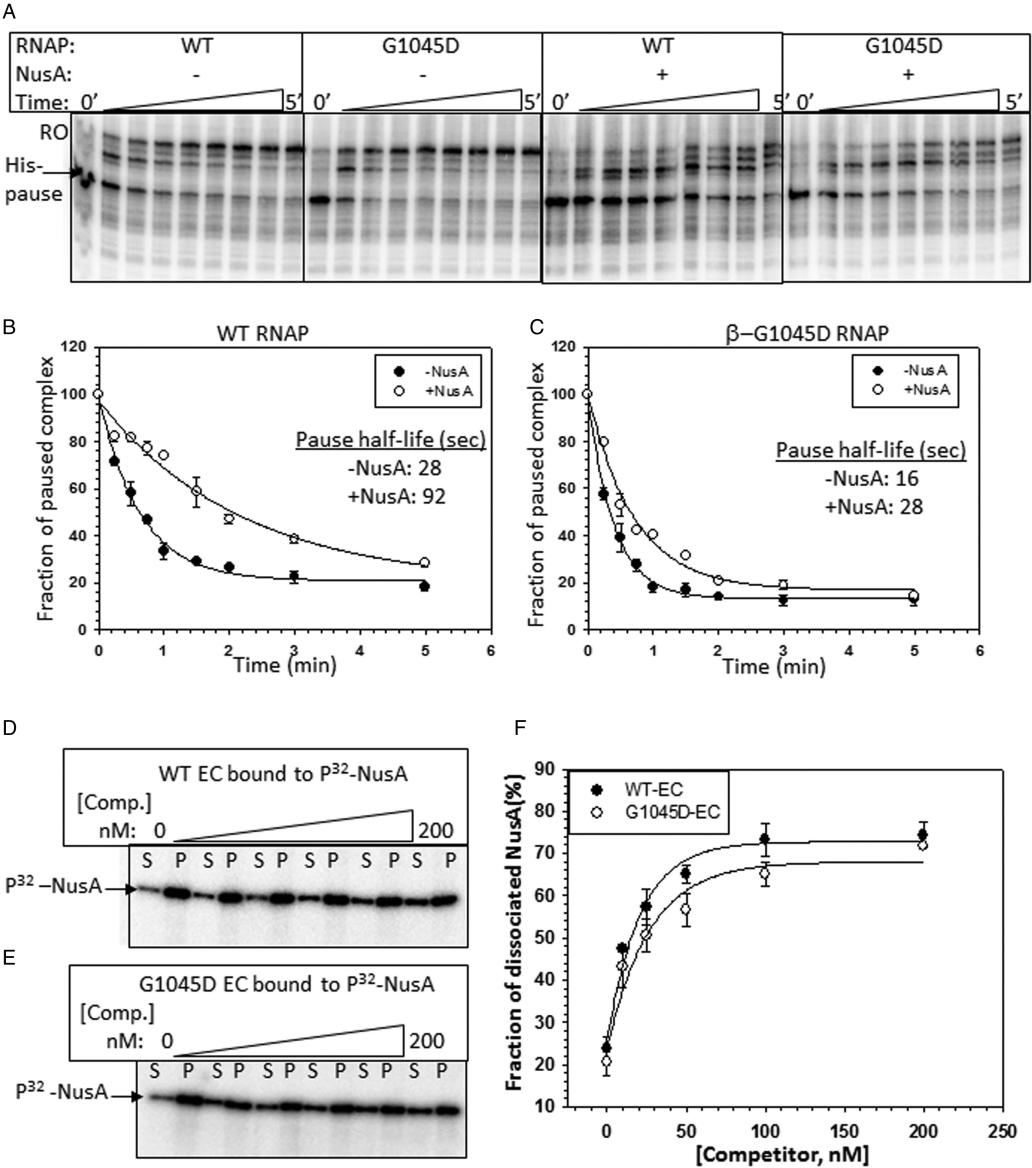
G1045D causes altered flap domain-NusA interaction. (**A**) Autoradiogram showing the pausing kinetics of WT and G1045D RNAP at the *his* pause site both in the presence and absence of NusA. RO denotes run-off product. (**B and C**) Fractions of paused complexes under different conditions were plotted against time to calculate the pausing parameters. Pause half-lives are indicated in the plots. (**D and E**) Autoradiogram showing the dissociation of radiolabeled NusA from the stalled ECs formed by WT and G1045D RNAP in the presence of increasing concentrations of unlabeled NusA as the cold competitor. S and P denote the supernatant and pellet fractions, respectively. (**F**) Fractions of dissociated P^32^-NusA {[2S] / ([S] + [S + P])} were plotted against increasing concentration of cold NusA. Data were obtained from (D) and (E). Errors were calculated from two to three measurements.

**Figure 4.**
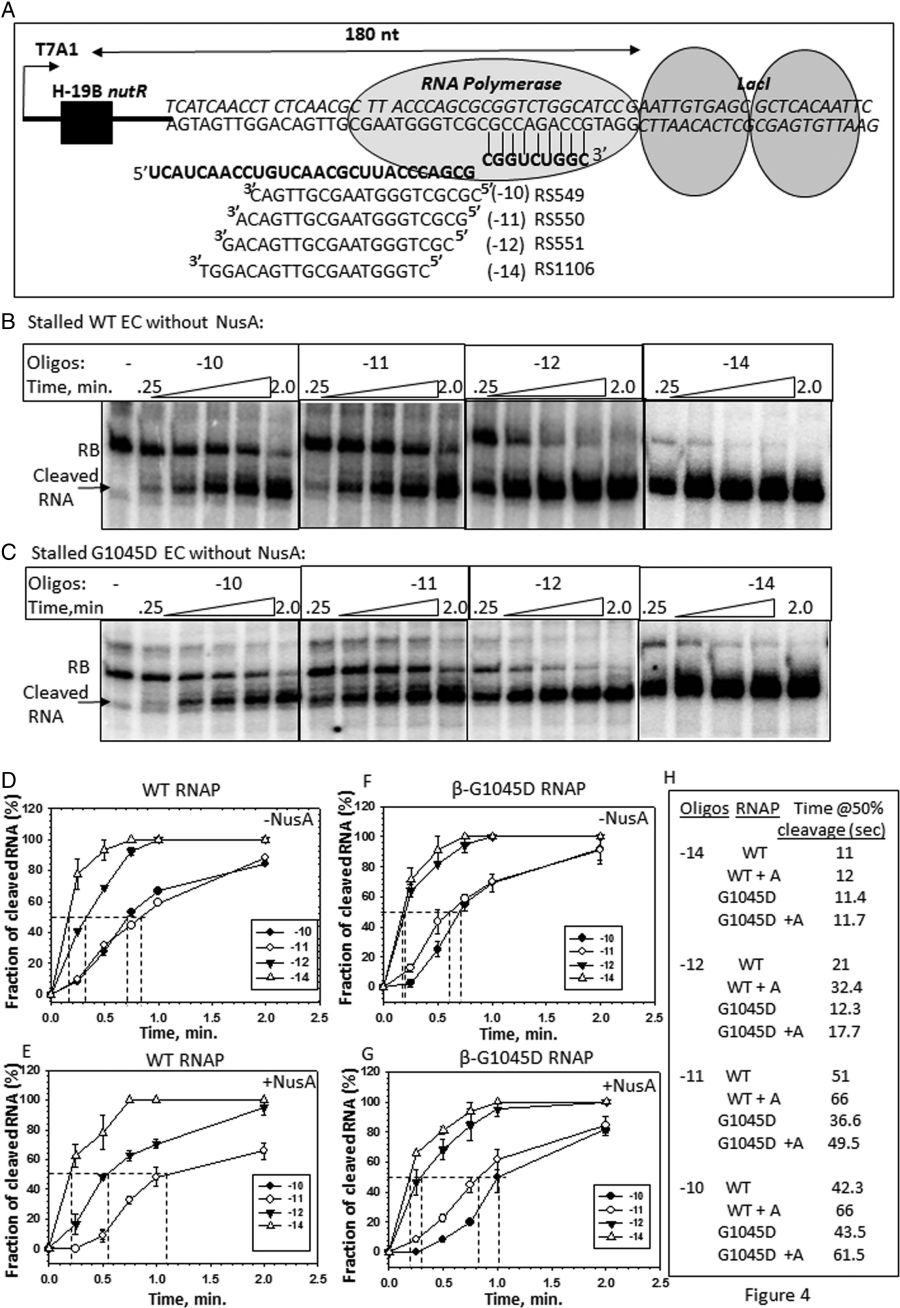
Alterations in the RNA exit channel by G1045D mutation. (**A**) Schematic showing the design of the stalled ECs formed by WT and G1045D RNAP and the sequences of the indicated antisense oligo-nucleotides (RS549, 550, 551 and 1106). Alignment of these oligos indicated the hybridization sites. −10, −11, −12 and −14 are the positions of the exiting RNA that correspond to the 5′-end of the antisense oligos. Stalled EC was formed 180 nt downstream of the *nut* site. (**B and C**) Autoradiograms showing the time course of RNase H cleavage profiles of the nascent RNA in the stalled ECs made by WT and G1045D RNAP in the absence of NusA. Antisense oligos in different panels are indicated. Cleaved and uncleaved RNAs are also indicated. (**D**–**G**) Plots showing the accumulation of the fractions of cleaved RNA ([cleaved RNA]/[Total RNA]) with time obtained in the presence of indicated antisense oligos. Please note that the curves corresponding to −10 and −11 oligos of (E) overlapped and appeared as a single curve. Vertical dotted lines on the time axes denote the time taken to achieve 50% RNA cleavage those are tabulated in (**H**). Errors were calculated from two to three measurements.

### Cleavage of stalled ECs with Fe-BABE conjugated NusA

Fe-BABE (p-Bromoacetamidobenzyl-EDTA) is a cleaving agent that can be specifically conjugated to a cysteine residue of a protein. Under reducing conditions (the reaction is initiated by addition of Ascorbic acid and H_2_O_2_), this reagent generates hydroxyl radicals. Three single Cys derivatives of NusA; S29C, S53C (in the NusA NTD domain) and S251C (in the SKK domain of NusA) were chosen to be conjugated to Fe-BABE.

In order to remove DTT and trace amounts of metal ions, 50 μl NusA (25 μM) protein was first dialyzed against metal removing buffer (30 mM MOPS, pH 8.2, 4 mM EDTA) and then against the conjugation buffer (30 mM MOPS, pH 8.2, 100 mM NaCl, 1 mM EDTA and 5% glycerol). 0.4 mM of Fe-BABE (Pierce) was incubated with 20 μM NusA in the conjugation buffer for 1 hr at 37°C. Unconjugated Fe-BABE was removed by passing the reaction mixture through a desalting column followed by equilibrating in the storage buffer (10 mM Tris-HCl (pH 8.0), 100 mM NaCl, 10% glycerol and 0.1 mM EDTA). Efficiency of Fe-BABE labeling was estimated from its sensitivity to DTNB (24).

HMK (Heart Muscle Kinase)-tagged RNAP (either rpoB or rpoC) were radiolabeled using protein kinase A and [γ-^32^P]ATP (3000 Ci/mmole). RB was formed in a similar way on the Template VII (T7A1-H-19B *nutR/t_R1_-lacO-T_R′_*) for RNase H cleavage assays, except that RNAP (either β or β′) was radiolabeled instead of RNA. 600 nM Fe-BABE-NusA either in the presence or absence of 600 nM N were incubated with the RB at 37°C for 10 min in the cutting buffer (50 mM MOPS, pH 8.0, 0.1 mM EDTA, 120 mM NaCl, 10 mM MgCl_2_ and 10% glycerol) prior to initiation of the cleavage reaction. Cleavage was initiated by adding ascorbate and hydrogen peroxide (final concentrations were 6 mM each) and the reaction was stopped by addition of one volume of 6X SDS sample buffer after incubation at 37°C for 5 min. Samples were heated to 95°C for 4 min prior to loading on 8% SDS-PAGE. Gels were exposed overnight to a phosphorimaging screen and the bands were analyzed in a Phosphorimager (Fuji).

For Fe-BABE-NusA mediated cleavage of nascent RNA in the RB, all the procedures were same as described above, except that RNA was radiolabeled during the formation of EC_23_ instead of radio-labeled RNAP.

## RESULTS

### Hypotheses

In our previous study (19), we have shown that during the process of the antitermination, N interacts with the convex surface of NusA-NTD that is located opposite to its RNAP-β-flap binding domain (Supplementary Figure S2). And hence, we hypothesized that one of the way of achieving the antitermination by N would be via allosterically modulating the NusA-β-flap interaction. N induced changes in the NusA-flap interaction may comprise of enhanced stability of this interaction as well as conformational changes of the flap domain through its spatial reorientation. We tested these hypotheses by (i) measuring the stability of the NusA-EC interaction; (ii) establishing the functional involvement of flap-domain in N antitermination; and (iii) probing the spatial orientations of the flap domain as well as the conformational changes in the RNA exit channel. In most of the experiments, N protein from the lambdoid phage H-19B was used. N protein from λ phage was used in Figure 1.

### N increases the affinity of NusA for the nut site but not for the RNAP of the EC

NusA has two modes of interactions with the EC; concave surface of NTD binds to the β-flap domain of RNAP and its SKK domain interacts with the nascent RNA (Supplementary Figure S1; (12)). With the *nut* site containing RNA, SKK domain makes specific contacts with the spacer region of this site (Supplementary Figure S1E and F). To test whether N increases the affinity of NusA for the EC, we measured the dissociation of radiolabeled NusA (P^32^-NusA) protein bound to the ECs stalled either close to the *λ nutR* site (∼60 nt downstream; Figure 1A, left panel; Template I) or away from it (∼250 nt downstream; Figure 1A, central panel; Template II) or from downstream of a terminator, *trpt’* that is devoid of *nut* site (Figure 1A, right panel; Template III) by ‘cold’ competition assays. These assays were performed both in the presence and absence of the N protein from the λ phage.

We used ∼10-fold molar excess of cold (non-radiolabeled) NusA as competitor, to each of the stalled ECs. The amounts of dissociated P^32^-NusA were measured from the supernatant fractions (S) (Figure 1B–E). We observed the following. (i) In the absence of λN, the NusA bound to the EC stalled on all the templates was competed out readily by the competitor (2^nd^ group of vertical bars in Figure 1E). (ii) In the presence of λN, this dissociation was significantly less (∼2-fold; compare the + and – N bars in Figure 1E) from the EC stalled on Template I. On this template, N decreased the dissociation of NusA even in the absence of the cold competitor. (iii) Even in the presence of λN, the dissociation of the radiolabeled NusA was higher from the EC stalled on Template II (away from *nut* site). (iv) This N-effect was absent when the P^32^–NusA was part of the EC stalled on Template III (devoid of *nut*site). These data indicated that the N-induced enhanced stability of NusA for the EC was dependent on the presence of *nut* site in the vicinity.

To further confirm the requirement of the *nut* site, we measured the dissociation of P^32^-NTD domain of NusA from the EC stalled on Templates I and III, both in the presence and absence of N. NusA-NTD interacts with RNAP but not with the nascent RNA (12). We observed that N was unable to enhance the stability of NusA NTD-EC complex (Supplementary Figure S3A and B, Figure 1F). Therefore, we concluded that the N-induced enhanced affinity of NusA for the EC was due to the higher affinity of the SKK domain of NusA for the spacer region of the *nut* site. It is likely that the interaction of ARM region of N with the adjacent *boxA* hairpin influences the NusA-spacer binding.

Results obtained above using both the full-length and the NTD fragment of NusA suggested that the stabilization effect of N on NusA-EC interaction is predominantly due to the increase of the affinity of NusA-SKK domain for the *nut* site. On the other hand, N is likely to influence the NusA NTD-β flap domain interaction by inducing altered conformational changes.

### RNAP β-subunit flap domain mutants have specific defects for N antitermination

Earlier, we have isolated a RNAP β-subunit flap domain mutant, G1045D, together with β′-subunit mutants located in and around the RNA exit channel (P251S, P254L and R270C) that were defective for N-mediated antitermination (21). In the same study, a suppressor, L108F, in the CTD (the RNAP-binding domain) of H-19B N was also found to specifically suppress the defects of the RNAP β′-subunit mutants. Interestingly, it was unable to suppress the antitermination defect of G1045D mutant. Also in the model structure of the EC of the *E. coli* RNAP (Figure 2A), G1045D bearing part of the flap-domain is located away from the exit channel, which is the proposed area through where N approaches the interior of the EC (25). Therefore, G1045D mutation most likely does not directly affect the binding of C-terminal domain of the N protein to the RNAP.

As NusA binds to the β-flap domain, we hypothesized that defect of N-mediated antitermination of the β flap mutant might be NusA mediated. To have a more detailed analysis of the involvement of β-flap domain in N antitermination as well as the role of NusA in the process, we engineered two more mutations in the β-flap domain, P1044A and V1046E (Figure 2A). We also included β V839E and R1058L mutations in the nearby region those were earlier reported to be defective for Q-mediated antitermination (26). P1044A and V1046E mutants were not viable at 42°C (Supplementary Figure S4A), so we did not perform *in vivo* antitermination assays. We performed *in vitro* antitermination assays with all the β flap mutants using a DNA template (Template IV) where H-19B *nut* site is cloned upstream of a triple terminator cassette, *T_R′_-T1-T2* (Figure 2B; Cheeran et al., 2005). The *in vitro* antitermination efficiencies (%RT) of H-19B N were measured from the signal of the run-off (RO) products from the autoradiograms (Figure 2B). G1045D, P1044A and V1046E RNAP mutants exhibited significant defects in N-mediated antitermination of the triple terminator cassette. G1045D was the most defective (Figure 2B and C). However, this defect was moderate for the mutants, P1044A and V1046E, when antitermination (%RT) was measured after the first terminator, *T_R′_*, which indicates that they are defective in processive antitermination. We also tested R1058L and V839E mutants in the same assays and observed that unlike Q antitermination, they were less defective for H 19B N mediated antitermination (Supplementary Figure S4B–D). Isolation of three flap domain mutants defective for N antitermination further reinforce the proposition that this domain of β-subunit of the RNAP is involved in this process.

If the hypothesis of N protein influencing NusA-flap domain interaction by the virtue of its interaction with NusA-NTD is correct, it is possible that these flap mutants may affect this N-induced modulations of the NusA-flap interaction. Therefore, it is expected that this β-flap mutant, G1045D, would exhibit a synthetic defect together with the NusA mutant, V8A that is defective for N-interaction, and is located in the N-NusA interaction surface (19). We observed that G1045D RNAP in the presence of WT NusA and V8A NusA in the presence of WT RNAP were partially defective for H 19B N-mediated antitermination at the *T_R′_* terminator. But when both V8A NusA and G1045D RNAP were expressed together in a strain (RS1452), the combination was severely defective for antitermination, thus exhibiting a strong synthetic defect (Figure 2E).

Results from aforementioned *in vitro* and *in vivo* studies strongly indicated that NusA-flap domain interaction is a component of the N antitermination process. We propose that N-NusA interaction modulates the NusA-flap interaction, and the flap mutants described above impair this modulation process.

### β-flap domain mutants affect flap domain-nascent RNA hairpin as well as NusA-flap interactions

Next, we wanted to understand the molecular basis of N-antitermination defect of the flap mutants. We have chosen G1045D as a prototype mutant as it was most defective for N-dependent antiterminaiton.

It has been established that the interaction of NusA and the β flap-tip helix (β 900–909 amino acids), located at the outer ridge of the RNA exit channel (Figure 2A), is the molecular basis for hairpin-dependent pause enhancement by NusA (27). To check whether G1045D mutation affects the NusA-flap interaction, we followed the pausing kinetics of WT and G1045D RNAP through the *his* pause site cloned downstream of the H-19B *nutR* sequence (Supplementary Figure S5A, Template VI). We found that G1045D mutation reduced the half-life of pausing significantly (from t_1/2_ = 28s to 16s, Figure 3A–C), which is similar to that observed upon flap-tip helix deletion (28). Interestingly, the G1045D mutation also caused reduced effect of NusA on the *his* pause enhancement (< 2-fold compared to > 3-fold for the WT RNAP) (Figure 3A–C). The pausing defect of G1045D mutant was specific for the *his* pause, as it did not exhibit significant defect at a hairpin-independent *ops* pause (Supplementary Figure S5B–D; Template V).

Reduction in pause half-life both in the absence and presence of NusA indicated that the G1045D mutation caused defect in the *his* pause hairpin-flap domain as well as the NusA-flap domain interactions. As G1045D mutation is located away (∼40Å) from the flap tip-NusA interaction site, it might have affected this interaction allosterically.

This pausing defect of G1045D RNAP might have arisen from its weaker interaction with the NusA-NTD. To test this, we measured the dissociation of P^32^-NusA from the ECs made either of WT or of G1045D RNAP stalled at the immobilized Template III DNA in the same way as described in Figure 1. Stability of NusA-EC complex was measured by competition assays using increasing concentrations of the ‘cold’ NusA as competitor (Figure 3D and E). Compared to its WT counterpart, we observed that for G1045D-EC required only slightly higher amount of the competitor to induce 50% dissociation of the labeled NusA (Figure 3F), which suggested that G1045D does not have significantly different affinity for the NusA-NTD.

Therefore, the G1045D mutation might have affected the flap-domain dynamics. And hence, we propose that (i) the flexible flap-tip might have undergone spatial re-orientation in the G1045D mutant; and (ii) the nature of flap domain-NusA interaction is also changed in this mutant. These altered conformations of the flap domain affected the interactions with the *his* hairpin, which reduced the pause half- life. As this mutation does not affect intrinsic termination properties (21), these changes in flap domain conformations are not enough to hinder the terminator hairpin formation in the RNA exit channel.

### G1045D mutation has altered RNA exit channel conformations

The structural model of the EC ((29); Figure 2A) revealed that the flap-domain forms one wall of the RNA exit channel and the flap-tip region comes within cross-linking distance of the exiting RNA at the outer ridge of the exit channel (Figure 2A; (27)). So it is possible that G1045D mutation might have changed the RNA exit channel conformatons. To test this, we have probed the conformational changes of the RNA exit channel by assessing its accessibility to form RNA:DNA hybrid with the antisense oligos having complementory sequences.

We stalled an EC 180 nt downstream of the *nut* site using lac repressor as a road block (Figure 4A). In this EC, the 3′-end of the growing RNA chain is 3 to 4 nucleotides upstream of the operator sequence, and the EC can be chased out from this position efficiently (30), which suggests that its configuration is in either pre- or post-translocated states. However, the presence of the repressor molecules at the downstream of the EC could affect RNAP clamp-domain movement which in turn could influence RNA:DNA hybrid formations in the RNA exit channel (31). As we are emphasizing on the relative differences between the RNA exit channel configurations of the WT and mutant RNAPs or between the presence and absence of N protein (described in the subsequent section) in the same ECs, this presumed alterations in the RNA exit channel due to hindered clamp domain is not likely to affect our results and interpretations.

We designed antisense DNA oligos whose 5′ ends correspond to the -10 (RS549), -11 (RS550), -12 (RS551) and -14 (RS1106) (Figure 4A) positions of the nascent RNA (where -1 position of the RNA is at the active site). These oligos can form RNA:DNA hybrids inside the RNA exit channel of stalled ECs made of either WT or G1045D RNAP (Figure 4A). Extent of formation of the hybrids as well as the overall accessibility of the channel was assessed by efficiency of RNase H cleavage of the RNA:DNA hybrids formed by these antisense oligos. In these experiments, kinetics of RNAseH cleavage actually reflects the rate of RNA:DNA hybrid formation and/or RNA digestion. Stalled ECs were formed both in the presence and absence of NusA. We have checked that these oligos did not induce oligo-mediated RNA release from the stalled ECs (Supplementary Figure S6A). RNase H mediated cleavage profiles and the plots of fractions of cleavage against time for different combinations are shown in Figure 4B and C and Supplementary Figures S6B, S6C and 4D-G, respectively. Time taken by RNase H to achieve 50% cleavage in each case was calculated from the plots and is tabulated in Figure 4H.

We observed the following. (i) In all the cases, RNA:DNA hybrid formed by -10 and -11 oligos were least accessible to RNase H cleavage, whereas that formed by -14 oligo was the most sensitive to the cleavage. This is consistent with the configuration of the exit channel, where -14 oligo is likely to form the hybrid at the outer edge of the channel (Figures 2A and 4A). (ii) In the NusA-bound WT EC, the RNase H accessibility was reduced for all the RNA:DNA hybrids except that formed by -14 oligo. The presence of NusA in the nearby flap-tip (see Figure 2A) region might have hindered the approach path of the enzyme or the channel is constricted. (iii) In the G1045D EC, the RNA:DNA hybrids formed by -11 and -12 oligos were more accessible to RNase H compared to its WT counterpart, indicating the presence of a more ‘widened’ exit channel. (iv) Effects of NusA on the exit channel dimension of this mutant EC were comparable with that for WT.

Therefore, G1045D mutation induces conformational changes in the RNA exit channel (Figure 4) as well as re-orientates the flap domain via altered NusA-flap interactions (Figure 3). As this mutation causes severe N-mediated antitermination defect, we hypothesized that N-modification of EC should involve ‘opposite’ types of conformational changes in the exit channel and in the flap domain. We tested this in the subsequent sections.

In this context, it should be noted that even though the reduced pausing exhibited by the β-G1046D RNAP is similar to that by N protein, the mechanism does not involve hindrance of pause hairpin formation in the exit channel (which is the case for N). Most likely the pause-reduction has occurred as a consequence of the altered NusA-flap tip interactions due to the *rpoB-*G1045D change.

### RNA exit channel becomes constricted in the presence of N and NusA

**Figure 5.**
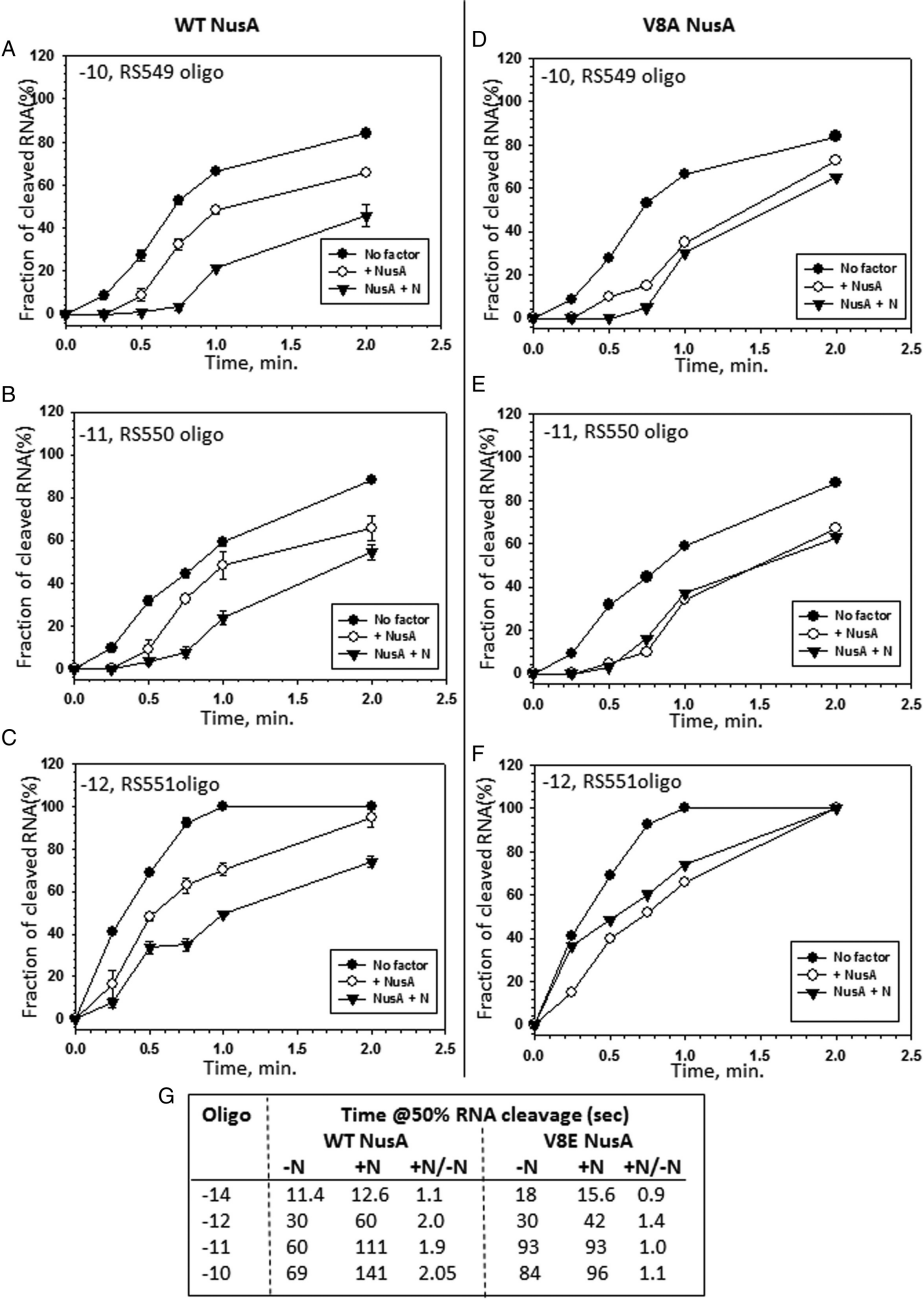
Kinetics of RNase H cleavage profile in the presence and absence of N. RNase H cleavage kinetics of the nascent RNA in the stalled ECs made with either WT (**A–C**, left side graphs) or V8E (**D–F**, right side graphs) NusA. ECs were made with WT RNAP and wherever indicated H-19B N was used. Experiments were done in the same way as described in Figure 4. Time required to obtain 50% cleavage in each case was obtained in the same way as in Figure 4 and is tabulated in (**G**).

We repeated the same RNase H cleavage assays (as in Figure 4) with the stalled EC made of WT RNAP in the presence and absence of H-19B N. We compared the effect of N in the presence and absence of WT NusA (Figure 5A-C, G, Supplementary Figure S7A and C). As N is unable to exert the antitermination in the presence of the NusA mutant, V8E (19), we used this mutant as a control (Figure 5D–F, G, Supplementary Figure S7B and D). We observed that the presence of N induced 2-fold slower rate of RNase H cleavage of the RNA:DNA hybrids formed by all the oligos, except that formed by the -14 oligo (Supplementary Figure S7C), and this N effect was not that significant in the presence of V8E NusA. It was also observed that V8E NusA alone exerted slightly higher protection from RNAseH. However, this mutant on its own did not have pausing (Supplementary Figure S8A) or termination defects (Supplementary Figure S8B; (19)), so this less sensitivity for RNAseH may not be functionally important.

We concluded the following from the above results. (i) N enhances the NusA mediated constriction of the exit channel. (ii) Unlike Q (32), N did not extend a NusA-mediated shield beyond this channel. We propose that this enhanced constriction of exit channel might have occurred due to N-induced altered NusA-flap tip as well as NusA-nascent RNA interactions, which presumably placed the flap-tip helix more towards the inside part of the channel from its natural location at the outer ridge (Figure 2; (29)). The presence of N-CTD in the exit channel (21,25) further reduces space in the channel, which might have also hindered the accessibility of the enzyme RNaseH. It is interesting to observe that even though the presence of NusA alone and together with N, both induce constriction of the exit channel, NusA alone stimulates termination and hairpin-dependent pausing while together with N causes antitermination and antipausing. We think this opposite outcome is due to N-induced altered NusA-flap domain interaction.

### Cleavage pattern of RNAP and nascent RNA by Fe-BABE conjugated to NusA-NTD changes in the presence of N

The concave surface of the NusA-NTD contacts with both the RNAP-flap and the nascent RNA (33). So we decided to probe the nature of the N-induced changes in the interaction patterns of NusA-NTD with the RNAP and the nascent RNA. We compared the changes in cleavage patterns of the exiting RNA as well as the RNAP obtained by OH-radicals emanated from the Fe-BABE moiety conjugated to different parts of NusA in the presence and the absence of N. This moiety can be conjugated to the cys residues, and upon activation by hydrogen peroxide and ascorbic acid, it is capable of generating OH-radicals that cleave nucleic acid or protein located within a radius of ∼12 Å of the labeled Cys residues (Supplementary Figure S9A). And hence, the cleavage pattern defines the regions surrounding the Cys residues.

**Figure 6.**
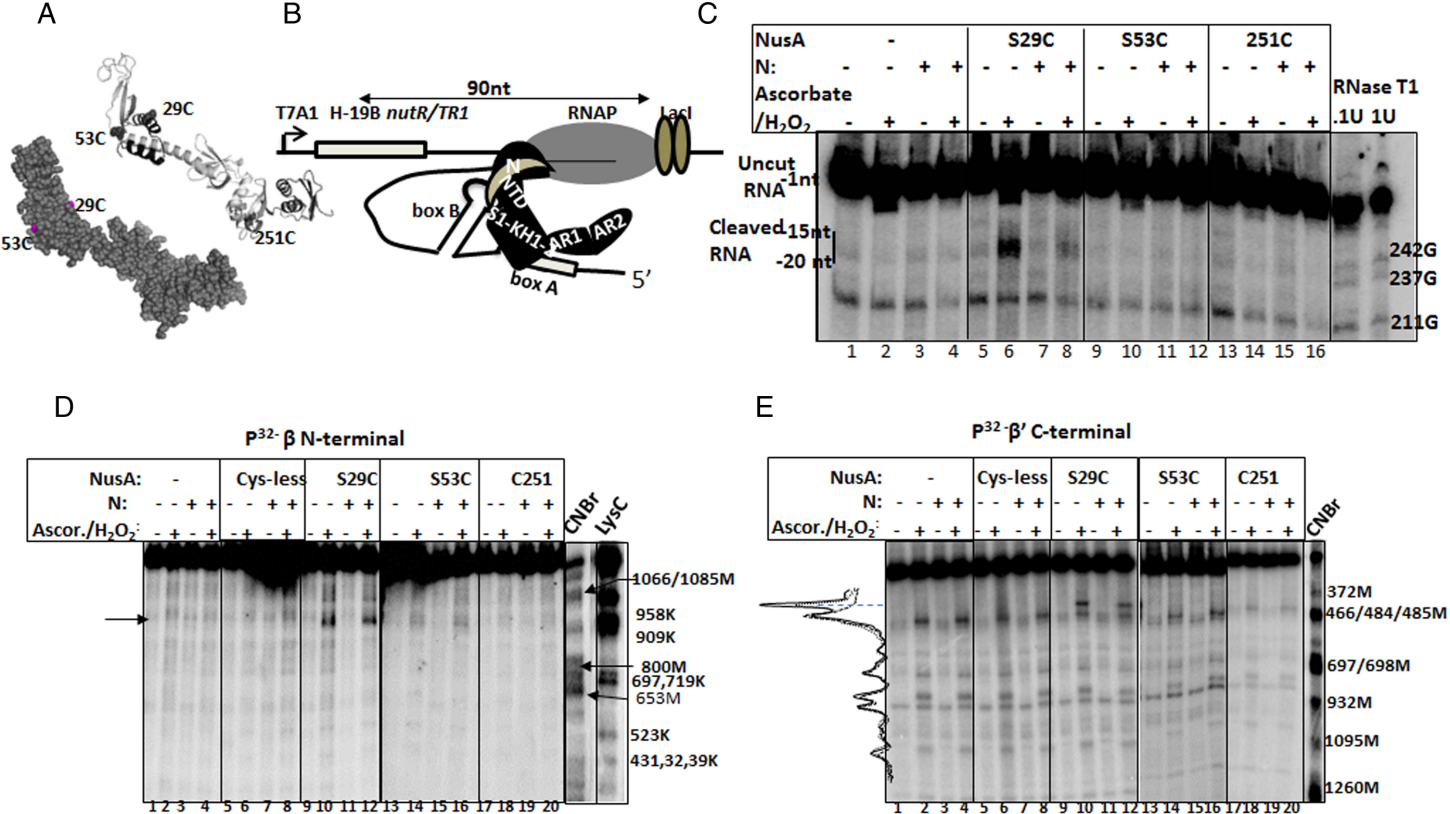
Alterations in the nascent RNA-β flap- NusA interactions in the presence of N. (**A**) Locations of single cys on the structure of NusA. These cys residues were labeled with Fe-BABE. (**B**) Schematic showing the composition of the stalled EC used for Fe-BABE cleavage assays. EC is modified with H-19B N and NusA having different single cys residues. (**C**) Autoradiogram showing the cleavage pattern of nascent RNA from the Fe-BABE moiety conjugated to different single cys derivatives of NusA under various conditions. Uncut and the cleaved RNAs are indicated. RNase T1 sequencing ladder is shown adjacent to the autoradiogram. Numbering of the G residues is according to the sequence of the nascent RNA. Position of the cleaved RNA is denoted with respect to the 3′-end of the nascent RNA, where the 3′ most residue is −1. (**D and E**) The autoradiograms of the Fe-BABE cleavage patterns of radiolabeled β and β′-subunits of the RNAP. Cleavage positions were mapped by comparing the sequencing ladder generated from the partial digestion by CNBr (for Met) and LysC (for Lys). The calibration plots are shown in Supplementary Figure S10. The curves adjacent to the gels show the intensity profile of the relevant lanes. Dashed, dotted and solid curves correspond to the lanes 4, 10 and 12, respectively, of (E). Cleaved bands are indicated. Ascor. means ascorbate.

We designed NusA derivatives with single Cys residues at the positions 29, 53 and 251 (Figure 6A), and conjugated each of them with Fe-BABE. Stalled ECs made of WT RNAP were formed with these Fe-BABE labeled NusA derivatives as described in Figure 6B. When required, these ECs were also modified with H-19B N. We monitored the Fe-BABE induced cleavage patterns of the nascent RNA (Figure 6C) as well the β (Figure 6D) and β′ (Figure 6E) subunits of the RNAP. In each experiments, first 23 nucleotides of the nascent RNA or N-terminal of the β-subunit or C-terminal of the β′-subunit of the RNAP were labeled with P^32^. The Fe-BABE-conjugated NusAs were functionally active in the *in vitro* N mediated antitermination assays (Supplementary Figure S9B).

**Figure 7.**
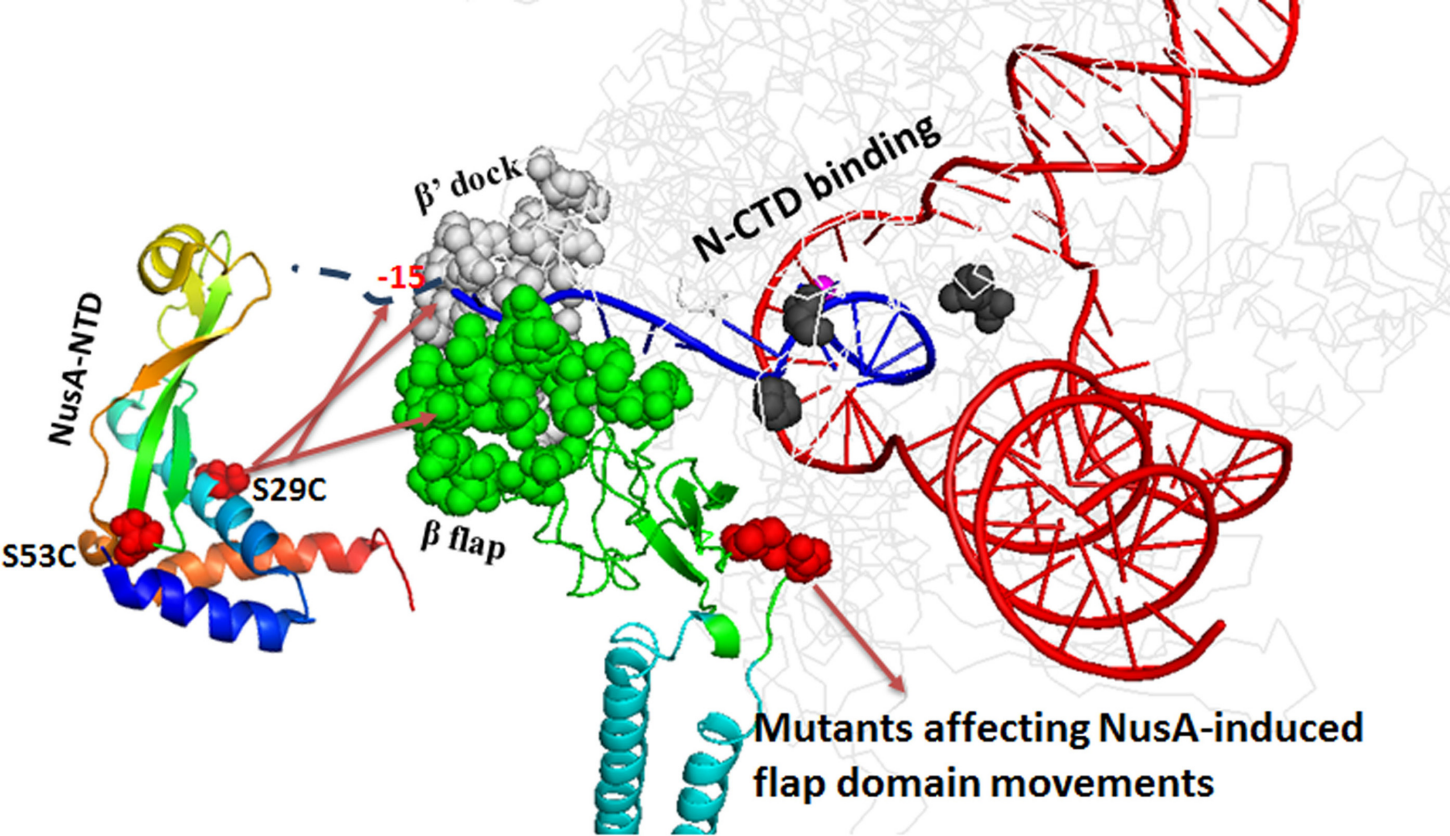
Model of transcription elongation complex (29) indicating the regions of β-flap and β′-dock domains affected due to the N-NusA interaction on the surface of the EC. The regions that were cleaved from S29C of NusA are indicated by arrows. DNA template and the emerging RNA are shown in red and blue, respectively. Part of RNA outside the exit channel is shown as dashed line. Flap domain is in green. The flap-tip helix is indicated as spheres on the ribbon diagram of flap domain. The dock domain is in gray. The flap domain mutants affecting the N-antitermination via hindering the domain movement are shown in red sphere. β′-mutants directly affected N-CTD binding (21) and are shown as black spheres inside the RNA exit channel. Active site Mg^+2^ in purple is also partially visible. Rest of the parts of the β and β′ subunits are shown in light gray in background.

When the stalled EC was formed with radiolabeled RNA and Fe-BABE conjugated NusA derivatives, we observed a broad cleaved band between -15 and -20 positions (where, -1 position is located at the active center) of the nascent RNA, only when the Fe-BABE conjugated S29C NusA was present (Figure 6C, lane 6). This region of the RNA maps just outside the exit channel (Figure 7). Broad signal indicates multiple cleavages at adjacent sequences. The intensity of the cleaved product significantly reduced when the EC was modified with N (lane 8). This cleavage was quite specific as it was not observed in the presence of either S53C (lanes 10 and 12) or 251C NusAs (lanes 14 and 16). S53C is located at the convex surface of NusA-NTD, opposite to its RNAP interaction surface. 251C is located in the RNA-binding domain that is away from the NusA-NTD. Also the cleaved products were absent in the absence of the initiator of the reactions, hydrogen peroxide and ascorbic acid (lanes 5, 7, 9, 11, 13, 15).

These results indicated that in the presence of N, either the concave surface having the 29C of NusA moved away from the exiting RNA or N itself protected this part of the RNA. We ruled out the second possibility because the RNA:DNA hybrid formed by the -14 oligo (Figure 5) was highly accessible to RNaseH cleavage even in the presence of N. So interaction of N at the convex surface of NusA-NTD induces changes in the orientation of the latter's concave surface, which is likely to change its site of interaction(s) on the β-flap domain.

Next we monitored the cleavage patterns of the radiolabeled β and β′ subunits of the RNAP present in the stalled EC in similar ways as described above (Figure 6D and E) and approximately mapped the Fe-BABE induced cleavage sites by comparing with the protein markers. We observed the following. (i) In the absence of N, specific cleavages at β (∼890 amino acid; Figure 6D, lane 10; Supplementary Figure S9A) and at β′ (∼ 410 amino acid; Figure 6E, lane 10; Supplementary Figure S9B) subunits were observed from the Fe-BABE conjugated to 29C-NusA. (ii) In the presence of N, the cleaved product from β-subunit was slightly retarded compared to that observed in the absence of N (Figure 6D, lane 12; Supplementary Figure S9). We did not consider this change significant as this might have arose from an artifact of gel electrophoresis. On β′-subunit, the cleavage intensity from 29C-Fe-BABE-NusA was significantly reduced (compare lanes 10 and 12 of Figure 6E and the intensity profiles). This indicates that the 29C residue of NusA might have moved away from this region of the β′-subunit. (iii) These cleavages were not observed from either Cys-less (lanes 6, 8 of Figure 6D and E) or 251C-Fe-BABe-NusA (lanes 19, 20 of Figure 6D and E) or in the absence of ascorbate and hydrogen peroxide (odd number lanes of Figure 6D and E), or in the absence of NusA (lanes 2, 4 of Figure 6D and E), which indicated that the cleavage patterns were specifically obtained from the Fe-BABE conjugated to NusA-NTD, the RNAP binding domain. (iv) It should also be noted that no cleavages on the RNAP subunits were obtained from the 53C-NusA that is located at the opposite side of the NusA-NTD RNAP binding surface (lanes 14 and 16 of Figure 6D and E).

These cleavage sites are located close to the β flap-tip helix (890–910 amino acids) and in the β′ dock domain (381–414 amino acids), which form the two sides of the RNA exit channel (Figure 7). Cleavage from NusA-NTD close to the flap-tip is consistent with recently reported mutations in this region that are defective for NusA-binding (33). Less drastic N-induced changes in the cleavage pattern of the RNAP (Figure 6D and E) compared to that obtained for nascent RNA (Figure 6B) suggests shuttle changes in the NusA-flap interaction pattern. Both these changes in the cleavage pattern together with that observed for the β′ dock domain arose due to N-induced reorientation of the concave surface of the NusA-NTD. However, this shuttle change is amplified into a significant spatial rearrangement of the β flap as well as the β′ dock domains to form a more constricted RNA exit channel in the N-modified EC (Figure 5).

## DISCUSSION

N-NusA interaction is the most important component in the N antitermination process (7). Till date it is not clear how this interaction converts the transcription elongation factor, NusA, into a facilitator of the antitermination function of N. In a previous report, we have shown that the functional interaction site of N on NusA is located at the opposite face of the NusA-NTD-β flap interaction surface (19). Here we have established that the N-NusA interaction alters the NusA-RNAP flap domain as well as NusA-nascent RNA interactions, which makes this transcription elongation factor to function as antiterminator. We have provided following evidences. (i) Mutations in the β-flap domain showed specific defect in N-antitermination (Figure 2), and one of them, G1045D, was shown to induce altered NusA-flap interaction (Figure 3). (ii) G1045D mutant also rendered a more accessible RNA exit channel (Figure 4 and Supplementary Figure S6). (iii) Consistent with the properties of the flap mutants, the presence of N induced an opposite effect on the WT RNAP by forming a lesser accessible and constricted exit channel (Figure 5 and Supplementary Figure S7). (iv) Altered Fe-BABE cleavage patterns of the RNAP and the exiting RNA from the concave surface of the NusA-NTD (Figure 6) strongly indicated N-induced reorientation of this RNAP-binding surface of NusA-NTD, which in turn might have changed the RNA exit channel dimensions by rearranging the β−flap and β′ dock domains (Figure 7). We propose that these N-NusA-induced changes in the conformations of RNA exit channel and the spatial reorientation of the β-flap and β′ dock domains are the two key elements that convert NusA into a facilitator of antitermination.

We have shown earlier that RNAP mutations in and around the RNA exit channel perturb N binding as well as its action (21)6 also in a separate study proposed that the N-CTD may penetrate into the active center of the EC through the RNA exit channel (25). Now we show that in addition to affecting these above regions of the EC, N also influences NusA to induce conformational changes of the β-flap domain, which in turn constricts the dimensions of the RNA exit channel.

N-induced antipausing (25) and antislippage (34) activities are indicative of its stabilization effect on the RNA:DNA hybrid (25,34). N has also been proposed to hinder the proper folding (21) or to delay the formation (3) of the terminator RNA hairpin in the exit channel. The flap domain connects to RNAP active site cleft through a two-stranded, antiparallel β sheet connector (Supplementary Figure S11), and it is possible that any movement in β-flap could affect the active site allosterically through this connector (27). And hence, N-induced altered NusA-NTD-flap interaction is likely to transmit signals to the active center through this path, which may induce antipausing as well as antislippage activities in the N-modified EC. On the other hand, constriction of the exit channel (Figure 5) in the presence of N could be the main reason for hindering terminator RNA as well as the *his* pause RNA hairpin formation in the channel.

Recent studies have revealed that targeting in and around the RNAP β-flap domain and modulating the RNA exit channel conformations are the common modes of action of different factors affecting the elongation and termination properties of the EC. The antiterminator, Q, binds to the β-flap domain of the RNAP and modulates the NusA-EC interaction in such a way that it forms an extended shield on the RNA exit channel (30,35,36). Recently, another antiterminator protein, gp39 from phage P23–45, has also been shown to target the β-flap domain (37). Its functional analog, p7 from the *X. oryzae* phage Xp10, binds RNAP near the β′-zinc-finger domain that forms the wall of the RNA exit channel located at the opposite side of the flap domain (37). The unique antiterminator, PUT RNA, also interacts with the same β′-zinc-finger (38,39).

RNA exit channel is the nucleation site for the terminator RNA hairpins and is presumably the approach path of the terminator, Rho, to invade the interior of the ECs (40,41). Therefore, in order to antagonize these terminator elements, it is quite logical for different antiterminators to be evolved to target the mobile and flexible regions, like the β-flap, β′-Zn-finger, etc., around the exit channel directly or via NusA to make the EC termination resistant. On the other hand, as the natural site of action of NusA is near the RNA exit channel (flp-tip helix), it becomes energetically favorable for the antiterminators to utilize it as its partner in the process of targeting the RNA exit channel.

## SUPPLEMENTARY DATA

Supplementary Data are available at NAR Online.

SUPPLEMENTARY DATA
