# Supplementary material for: N protein from lambdoid phages transforms NusA into an antiterminator by modulating NusA-RNA polymerase flap domain interactions
Source: Nucleic Acids Res. 2015 May 18;43(12):5744–58. doi: 10.1093/nar/gkv479 (PMC4499122; doi:10.1093/nar/gkv479)
Supplement: SUPPLEMENTARY DATA [file supp_43_12_5744__index.html]

N protein from lambdoid phages transforms NusA into an antiterminator by modulating NusA-RNA polymerase flap domain interactions — N protein from lambdoid phages transforms NusA into an antiterminator by modulating NusA-RNA polymerase flap domain interactions — SUPPLEMENTARY DATA 

# N protein from lambdoid phages transforms NusA into an antiterminator by modulating NusA-RNA polymerase flap domain interactions

## SUPPLEMENTARY DATA

- SUPPLEMENTARY DATA
