# Supplementary material for: N protein from lambdoid phages transforms NusA into an antiterminator by modulating NusA-RNA polymerase flap domain interactions
Source: Nucleic Acids Res. 2015 May 18;43(12):5744–58. doi: 10.1093/nar/gkv479 (PMC4499122; doi:10.1093/nar/gkv479)
Supplement: SUPPLEMENTARY DATA [file supp_gkv479_nar-00731-f-2015-File009.pdf]

Supplementary Methods

Materials.

NTPs were purchased from GE Healthcare. [γ-^{32}P]ATP (3000 Ci/mmol) and [γ-^{32}P]CTP (3000 Ci/mmol) were from Jonaki, BRIT, India. Antibiotics, lysozyme, DTT, and BSA were purchased from U. S. Biochemical. Primers for PCR were purchased either from Xcelris or MWG. HPLC pure antisense oligos used in the foot-printing experiments were from MWG. Restriction endonucleases, polynucleotide kinase, and T4 DNA ligase were from New England Biolabs. WT *E. coli* RNAP holoenzyme and RNAseH were purchased from Epicenter Biotechnologies. Taq DNA polymerase was from Roche Applied Science. Ni-NTA-agarose beads were purchased from Qiagen. Streptavidin-coated magnetic beads were purchased from Promega. Fe-BABE (p-Bromoacetamidobenzyl-EDTA), a protein cutting reagent, was obtained from Thermo scientific. All the bacterial growth media were from Difco. All the bacterial strains, plasmids, and oligos used are listed in Table S1.

Pausing assays and termination assays with WT and V8E NusA.

For pause assays, the templates with *T7A1-nutR-his pause* sequence was used. First, EC_{23} was made in a similar way as in other *in vitro* transcription assays. To determine the rate of elongation complex on the his pause template, the EC_{23} was chased in the presence of 150 µM of UTP, CTP, and ATP and 5 µM of GTP. Concentrations of different components used in this were as follows: 20 nM RNAP, 5nM template, 300nM WT or V8E NusA. Aliquots were removed during the time course (0, 15, 30, 60, 90, 120, 180, 240, 360, 480 s) and mixed with 2X loading ambion dye. RNA products were separated on denaturing 8% polyacrylamide gel. The pause half-lives were measured from the apparent pause escape rates ($t_{1/2}$=ln2/λ).

For the termination assays, single round transcription on the T7A1-nutR-$T_{R'}$ template was using following the standard protocols.

HMK-tagged RNA Polymerases, NusA and N proteins

Cloning and purifications of heart muscle kinase (HMK) tagged *E.coli* RNA polymerase are described elsewhere (Cheeran et al., 2007). Both HMK-tagged RNAPs were tested for their *in vitro* antitermination efficiencies with the H-19B N. Because the RNAP preparations were not fully saturated with sigma-70, the transcription reactions were supplemented with sigma-70. HMK-tags were introduced at the C- and N-terminals of different *E.coli* NusA derivatives by following the published procedures (Cheeran et al., 2007). The HMK tags were used to transfer P^{32} from [P^{32}] ATP (~3000Ci/mmol) onto β and β' subunits of the RNAP and the reaction is catalyzed by Protein Kinase A. H-19B and λ N proteins were purified following the published procedures (Cheeran et al., 2005).

N-terminal-HMK tagged G1045D, P1044A, V1046E, V839E and R1058L mutants of the RNAP β-subunit (*rpoB*) were made by site directed mutagenesis using pRS484 (Cheeran et al., 2007) as a template. These mutant RNAPs were purified following the published procedures (Kashlev et al., 1996).

Preparations of molecular weight markers used in Fe-BABE cleavage assays.

Molecular weight markers of end-labeled RNAP (either N-terminal HMK tag β or C-terminal β') were generated by cyanogen bromide (CNBr), Sub-maxillary protease (Arg-C) and Lysylendopeptidase (Lys-C) mediated cleavages.

Methionine specific cleavage reaction was performed using CNBr in a 10 µl reaction mixture containing 0.2 µM of labeled RNAP, 0.1 M CNBr and 0.4% (w/v) SDS (pH adjusted to pH 2 with 1 M HCl). At first, labelled RNAP was incubated at 37ºC for 10 min with 0.4% (w/v) SDS and cleavage reaction was started with the addition of 0.1 M CNBr for 5 min at 37ºC. Reactions were terminated by addition of 6X SDS-loading dye followed by boiling.

Arginine specific cleavage reaction was performed in a 10 µl reaction mixture containing 0.2 µM of labeled RNAP, 5 µg of Sub-maxillary protease and 0.1% (w/v) SDS. Reaction was started with the addition of 5 µg Arg-C after the incubation of the RNAP with 0.1% SDS at 37ºC for 10 min and stopped after 10 min by addition of 6X SDS-loading dye followed by boiling.

For the lysine specific cleavage, 0.2 µM of labelled RNAP was mixed with 6.33 µM urea in 50 mM Tris-Cl buffer and incubated at 37ºC for 10 min. The cleavage reaction was started with the addition of 50 ng Lys-C at 37ºC and stopped after 10 min by addition of 6X SDS-loading dye followed by boiling.

Legends to the supplementary figures:

Figure S1. **A)** Cartoon representing the lambdoid phage protein N (~12 kDa) that is intrinsically unstructured and attains structure upon binding with RNA, NusA and RNAP. Different functional domains are indicated. **B)** Cartoon showing different functional domains of 55-kDa NusA. The structural model of the *E. coli* NusA are shown below the cartoon. The NTD, S1, KH1 and KH2 domains were modelled using the program SWISSMODEL. AR1 (PDB ID 1WCL) and AR2 (PDB ID 1WCN) are from NMR structures of *E. coli* NusA. **C)** Cartoon represents the N-NusA interactions on the elongation complex according to Mah et al. 1999. **D)** The early region of lambdoid phages. The N gene is indicated. Antiterminated transcripts in the presence of N are indicated. These transcripts originating from P_L and P_R promoters bypass the tR and tL terminators in the presence of N. **E)** The components of the N binding site, nut site, on the mRNA. N recognizes the GNRA tera-loop of the boxB RNA hairpin. **F)** Cartoon showing an EC modified by N and other Nus factors.

Figure S2. **A possible model of the N-NusA NTD-EC ternary complex.** Exiting RNA(*red*),-flap (*dark gray*), and NusA-NTD (*cyan*) are highlighted. The rest of the β / β' are shown in *light grey*. N is shown as a cartoon. N and RNAP binding residues of NusA-NTD are in *red* and *green spheres*, respectively. The RNA outside the EC is shown as a dotted line.

Figure S3. A) and B) Autoradiogram showing half of the dissociated amount of radio-labelled NTD of NusA (^{32}P-NusA NTD) as S (half of supernatant) and the remaining part as P (half of supernatant + pellet). NusA was bound to the EC on the Templates I and III, respectively, in the presence and absence of N (100 nM) and the cold competitor NusA-NTD (100 nM).

Figure S4: Characterizations of *β flap mutants.* **A)** Viability assays to check the effects of *rpoB* mutants, V1046E and P1044A on the growth of the cells. RS860 strain was transformed with pHyd534 having *rpoB* either with the WT or mutant sequences. Plates were incubated either at 30 °C or at 42°C. Strains transformed with the plasmids having V1046E and P1044A mutations were not viable at 42°C. **B)** *In vitro* anti-termination assays on the same template as shown in figure 2B. RO denotes the run-off product. Concentrations of DNA template, RNA polymerase and NusA were 5 nM, 25 nM and 300 nM, respectively. Samples were run on a 6% sequencing gel and graphs (**C** and **D**) were plotted as read through (RO) at 3T v/s concentrations of N and read through (RO) at $T_{R'}$ v/s concentrations of N.

Figure S5: *Pausing activity of G1045D flap mutant*. **A)** Cartoon showing the NusA-modified EC stalled at the *his* and *ops* pause site using Lac repressor as a roadblock respectively. Pause sequence, pausing sites are indicated. **B)** Autoradiograms showing the time course of transcription elongation through the *ops* pause sequence (indicated by *arrow*) both in the absence and presence of WT NusA. Samples were removed at 0, 15, 30, 45, 60, 90, 120, 180 and 240 sec and were quenched. RNA products were separated in 8% denaturing polyacrylamide gel. Run-off product is indicated as "RO." **C) and D)** Fractions of paused complex obtained under different conditions are plotted against time. The plots were fitted to the equation of exponential decay using SIGMAPLOT to calculate the escape rates (λ) and the pause half-lives ($t_{1/2}$=ln2/λ).

Figure S6: A) Stalled elongation complexes formed on immobilized template were incubated with different anti-sense oligos (see figure 4A) at 37^0C for 10 min. RNA release was measured in the supernatant (S) fraction. **B**) and **C)** Autoradiograms showing the RNAse H mediated cleavages of the nascent RNA from the stalled ECs made of WT and G1045D RNAP in the presence of NusA. Different anti-sense oligos were indicated. Same DNA template was used as described in figure 4.

Figure S7: A) and **B)** Autoradiograms showing the time courses of RNAse H cleavage profiles in the presence under indicated conditions. Same stalled ECs were used as described in figure 4. **C)** and **D)** Fractions of cleaved RNA in the presence of -14 oligo were plotted against time in the presence of WT and V8E NusA.

Figure S8: A): V8E NusA stimulates pausing at the His-pause sequence. Autoradiogram showing the time course of transcription elongation through a *his* pause sequence both in the absence and presence of 300nM WT or V8E NusA mutant. Pause position and Run-off products are indicated. Aliquots were removed during the time course (0, 15, 30, 60, 90, 120, 180, 240, 360, 480 s). The pause half-lives ($t_{1/2}$) are indicated.
B) V8E NusA mutant enhances termination at TR' terminator. Autoradiogram showing the single round *in vitro* transcription termination in the presence and absence of either WT NusA or V8E

NusA on a template with the intrinsic terminator TR'. Concentrations of DNA template and RNA polymerase were 5 nM and 25 nM, respectively. Concentrations of NusA proteins are indicated.

Figure S9: A) Cartoon showing Fe-BABE moiety conjugated to 29C position of NusA. **B)** Transcription antitermination assays with the Fe-BABE conjugated NusAs. RO and $T_{R'}$ indicate run-off and terminated products, respectively. Amounts of read-through values (RT%) are also indicated.

Figure S10: To compensate for the sequence-dependent effects on the migration rates of the proteins on the SDS-PAGE, cleavage sites on β and β′ subunits were marked by comparison with sequence markers generated from the same protein. As the reference markers, β and β′ subunits were cleaved at either Met or Lys residues by chemical digestions of N terminal radiolabeled β or C terminal radiolabeled β′ to produce known marker fragments. The log molecular weight is plotted against relative migration distance (Rm) of the marker fragments on the SDS-PAGE. **(A)** β sequence markers. **(B)** β′ sequence markers. Curve fittings were done using an inverse third order polynomial equation. Cleavage sites are indicated.

Figure S11: The connector regions (in black), connecting flap domain (in green) with the active center (purple Mg^{+2} ion), are highlighted in the structure of EC. DNA template and exiting RNA are shown in red and blue, respectively.

References:

Kashlev, M., Nudler, E., Severinov, K., Borukhov, S., Komissarova, N. and Goldfarb, A. (1996) Histidine-tagged RNA polymerase of Escherichia coli and transcription in solid phase. *Methods in enzymology*, **274**, 326-334.

Cheeran, A., Babu Suganthan, R., Swapna, G., Bandey, I., Achary, M.S., Nagarajaram, H.A. and Sen, R. (2005) Escherichia coli RNA polymerase mutations located near the upstream edge of an RNA:DNA hybrid and the beginning of the RNA-exit channel are defective for transcription antitermination by the N protein from lambdoid phage H-19B. *Journal of molecular biology*, **352**, 28-43.

Cheeran, A., Kolli, N.R. and Sen, R. (2007) The site of action of the antiterminator protein N from the lambdoid phage H-19B. *The Journal of biological chemistry*, **282**, 30997-31007.

Mah TF, Li J, Davidson AR, Greenblatt J. (1999) Functional importance of regions in Escherichia coli elongation factor NusA that interact with RNA polymerase, the bacteriophage lambda N protein and RNA. Mol Microbiol. 34, 523-37.

Table S1. Strains, plasmids and oligos.

Strains	Description	Reference
RS860	MG1655, *rpoB (ts), tet* ^{R}	This study
RS1018	MC4100 *galEp3*, λRS45 lysogen carrying P_{lac}– H-19B *nutR*-t_{R1}- $T_{R'}$ *-lac ZYA*	This study
RS1452	RS1018 having *G1045D rpoB.*	This study
**Plasmids**		
pK8601	pGB2 with plac- H-19B N, *spec*^{R}.	(3)
pRS22	pTL61T with *pT7A1– H-19B nutR*-$T_{R'}$*-T1T2-lacZYA*, amp^R.	(3)
pRS25	pTL61T with *pT7A1– H-19B nutR (ΔcII)* $T_{R'}$*-T1-T2-lacZYA*, amp^R	(3)
pRS88	*rpoB* was cloned into BamH1/HindIII sites in pBR322	(3)
pRS106	pTL61T with *pT7A1-trpt'-lacZ, amp*^{R}	(4)
pRS385	pRS25 with *T7A1-nutR-lacO*-$T_{R'}$ fusion, amp^R.	(5)
pRS484	pTRC99A-*E. coli rpoB* with N-terminal HMK, His tag β S523Y-NPH *rpoB*, amp^R.	(5)
pRS513	pBAD18M- *E. coli rpoC* with C-terminal HMK, His tag, amp^R.	(5)
pRS523	WT NusA cloned at NdeI/XhoI site of pET33b, HMK, His tag at N- terminal, kan^R	(5)
pRS604	pTL61T with *pT7A1– λ nutR-T1T2-lacZYA*, amp^R..	This study
pRS615	*λ N* cloned at NdeI/XhoI site of pET21b, amp^R.	This study
pRS703	pHyd3011 having WT *nusA*, amp^R.	This study
pRS1100	pET33b having Cys-less NusA, HMK, His tag at N- terminal, kan^R	
pRS1101	pHyd3011 having *V8A nusA*, amp^R	This study
pRS1120	pET33b having C251 NusA, HMK, His tag at N- terminal, kan^R	This study
pRS1124	pRS1100 with S29C *nusA*, HMK, His tag at N- terminal, kan^R	This study
pRS1193	pRS1100 with S53C *nusA*, His tag at N- terminal, kan^R	This study
pRS1566	pRS484 with *rpoB* R1058L, amp^R	This study
pRS1567	pRS484 with *rpoB* G1045D, amp^R	This study
pRS1569	pRS484 with *rpoB* V1046E, amp^R	This study
pRS1570	pRS484 with *rpoB* V839E, amp^R	This study
pRS1571	pRS484 with *rpoB* P1044A, amp^R	This study
pRS1568	pRS88 with P1044A *rpoB*, amp^R	This study
pRS1572	pRS88 with V1046E *rooB*, amp^R	This study
**Oligos**		
RSRK1	CGCCAGGGTTTTCCCAGTCACGAC; reverse primer in the *lacZ* gene of pTL61T.	
RS2	CTTGCATGCCTGCAGGTCGACTC, downstream oligo after $T_{R'}$ on of *T7A1-nutR*-$T_{R'}$ of pRS25	
RS58	ATAAACTGCCAGGAATTGGGGATCG, forward primer of pTL61T	
RS83	Biotinylated RS58 forward primer of pTL61T (and all its derivatives like pRS106, pRS25) vector sequence.	
RS177	GTGAGCGCTCACAATTCGGATATATATTAACAATTACCTG, reverse primer having *lacO* sequence fusion at 161U of trpt' terminator, used with forward primer RS83 on pRS106	

RS263	CTGAAAGACTAGTCAGGATGATGGTTGGCCTTAGTTGGTCAGATATATTGGG; downstream oligo 1 for making *his* pause template, used for PCR amplification with forward primer RS83 on pRS25	
RS264	CTGAAAGACTAGTCAGGATGATGGTTGGCCTTAGTTGGTCAGATATATTGGG; downstream oligo 2 for making *his* pause template, used for PCR amplification with RS83 on template made from RS83/RS263	
RS265	CTGAATGTCTTCCAGCACACATCGCCTGAAAGACTAGTCAGGATGATGGTTG; downstream oligo 3 for making *his* pause template, used for PCR amplification with RS83 on the template made from RS83/RS264 pair	
RS267	TGCGTGGAAAAAACGCACGCTACCGCCTGGCCTTAGTTGGTCAG ATATATTGGG; reverse primer for making *ops* pause template, used for PCR amplification with forward primer RS83 on pRS25.	
RS275	GGAATTGTGAGCGCTCACAATTCCTTC*CA*GCACACATCGC CTGAAAGACTAG; reverse primer having *lacO* sequence to make road block (RB) at 4nt downstream of the *his* pause position, used for PCR amplification with RS83 on the template made from RS83/RS265 pair	
RS276	GGAATTGTGAGCGCTCACAATTCAAC*G*CACGCTACCGCCTGGCC; reverse primer to make RB after 3nt of ops pause position, used for PCR amplification with RS83 on the template made from RS83/RS 267 ops pause template	
RS333	GAATTGTGAGCGCTCACAATTCGGATGCAAATAAATGCATACACCA, *lacO* fusion at 60 nt downstream of *λ nutR* site	
RS404	GAATTGTGAGCGCTCACAATTCGGATGCCAGACCGCGC TGGGTAAGCG; reverse primer with *lacO* sequence at 180 nt downstream of *H-19B nutR*.	
RS542	CTTCGTCACCGAACCAGGAAATATTACGGGCAGC downstream oligo for SDM C489S NusA.	
RS549	CGCGCTGGGTAAGCGTTGAC, Antisense Oligo upstream of RS404 Roadblock positions of H-19B (5'-end of oligo correspond to the -10 position of nascent RNA)	
RS550	GCGCTGGGT AAGCGTTGACA, Antisense Oligo upstream of RS404 Roadblock positions of H-19B (5'-end of oligo correspond to the -11 position of nascent RNA)	
RS551	CGCTGGGT AAGCGTTGACAG, Antisense Oligo upstream of RS404 Roadblock positions of H-19B (5'-end of oligo correspond to the -12 position of nascent RNA)	
RS885F	GGGCGACGATCTGGCACCGGACGTGCTGAAGATTGTTAAGG, forward primer for SDM G1045D in *rpoB.*	
RS885R	CCTTAACAATCTTCAGCACGTCCGGTGCCAGATCGTCGCCC, reverse primer for SDM G1045D in *rpoB.*	
RS970	GGT ATA TCT GGC GGT TAA ACT CCG TAT CCA GCC TGG TGA; forward primer for SDM R1058L in *rpoB*	
RS971	GTC ACC AGG CTG GAT ACG GAG TTT AAC CGC CAG ATA TAC C; reverse primer for SDM R1058L in *rpoB*	
RS994	GAATTGTGAGCGCTCACAATTCTTAGCCTACTCTCGCATGGGGAGACC, *lacO* fusion at 250 nt downstream of *λ nutR* site	
RS 1013	CAC ATT CAG GAA CTG GCG TGT GAG TCCCGT GAC ACC AAG CTG GGT CCG; forward primer for SDM V839E in *rpoB*	
RS1014	CGG ACC CAG CTT GGT GTC ACG GGA CTC ACA CGC CAG TTC CTG AAT GTG; reverse primer for SDM V839E in *rpoB*	
RS1015	GAC GAT CTG GCA CCG GGC GUG CTG AAG ATT GTT AAG GTA; forward primer FP for SDM V1046E in *rpoB*	
RS1016	TAC CTT AAC AAT CTT CAG CAC GCC CGG TGC CAG ATC GTC; reverse primer for SDM V1046E in *rpoB*	

RS1017	CAG GGC GAC GAT CTG GCA GCG GGC GTG CTG AAG ATT GTT AAG; forward primer for SDM P1044A in *rpoB*	
RS1018	CTT AAC AAT CTT CAG CAC GCC CGC TGC CAG ATC GTC GCC CTG reverse primer for SDM P1044A in *rpoB*	
RS1106	CTGGGTAAGCGTTGACAGGT, Antisense Oligo upstream of RS404 Roadblock positions of H-19B (5'-end of oligo correspond to the -14 position of nascent RNA)	

References:

1. Muteeb, G., Dey, D., Mishra, S., and Sen, R. (2012) A multipronged strategy of an anti-terminator protein to overcome Rho-dependent transcription termination. *Nucleic acids research* **40**, 11213-11228.
2. Mishra, S., Mohan, S., Godavarthi, S., and Sen, R. (2013) The interaction surface of a bacterial transcription elongation factor required for complex formation with an antiterminator during transcription antitermination. *The Journal of biological chemistry* **288**, 28089-28103.
3. Cheeran, A., Babu Suganthan, R., Swapna, G., Bandey, I., Achary, M. S., Nagarajaram, H. A., and Sen, R. (2005) Escherichia coli RNA polymerase mutations located near the upstream edge of an RNA:DNA hybrid and the beginning of the RNA-exit channel are defective for transcription antitermination by the N protein from lambdoid phage H-19B. *Journal of molecular biology* **352**, 28-43.
4. Pani, B., Banerjee, S., Chalissery, J., Muralimohan, A., Loganathan, R. M., Suganthan, R. B., and Sen, R. (2006) Mechanism of inhibition of Rho-dependent transcription termination by bacteriophage P4 protein Psu. *The Journal of biological chemistry* **281**, 26491-26500.
5. Cheeran, A., Kolli, N. R., and Sen, R. (2007) The site of action of the antiterminator protein N from the lambdoid phage H-19B. *The Journal of biological chemistry* **282**, 30997-31007.

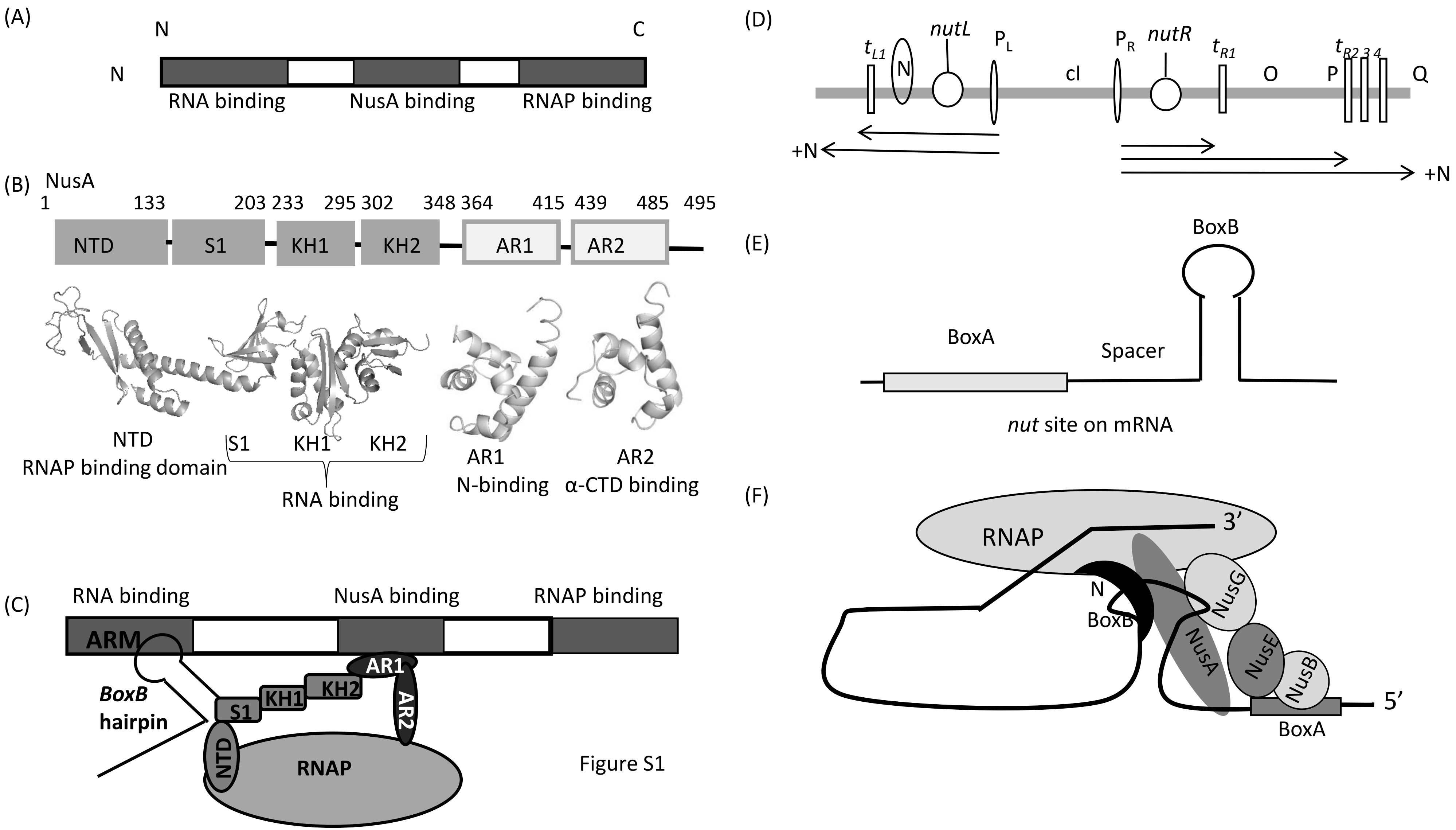

(A)
N
C
N
RNA binding
NusA binding
RNAP binding
(B)
NusA
1
133
203
233
295
302
348
364
415
439
485
495
NTD
S1
KH1
KH2
AR1
AR2
NTD
RNAP binding domain
S1
KH1
KH2
RNA binding
AR1
N-binding
AR2
α-CTD binding
(C)
RNA binding
NusA binding
RNAP binding
ARM
BoxB
hairpin
S1
KH1
KH2
AR1
AR2
NTD
RNAP
(D)
tL1
N
nutL
PL
cI
PR
nutR
tR1
O
P
tR2 3 4
Q
+N
+N
(E)
BoxB
BoxA
Spacer
nut site on mRNA
(F)
RNAP
3'
N
BoxB
NusA
NusG
NusE
NusB
BoxA
5'

Figure S1

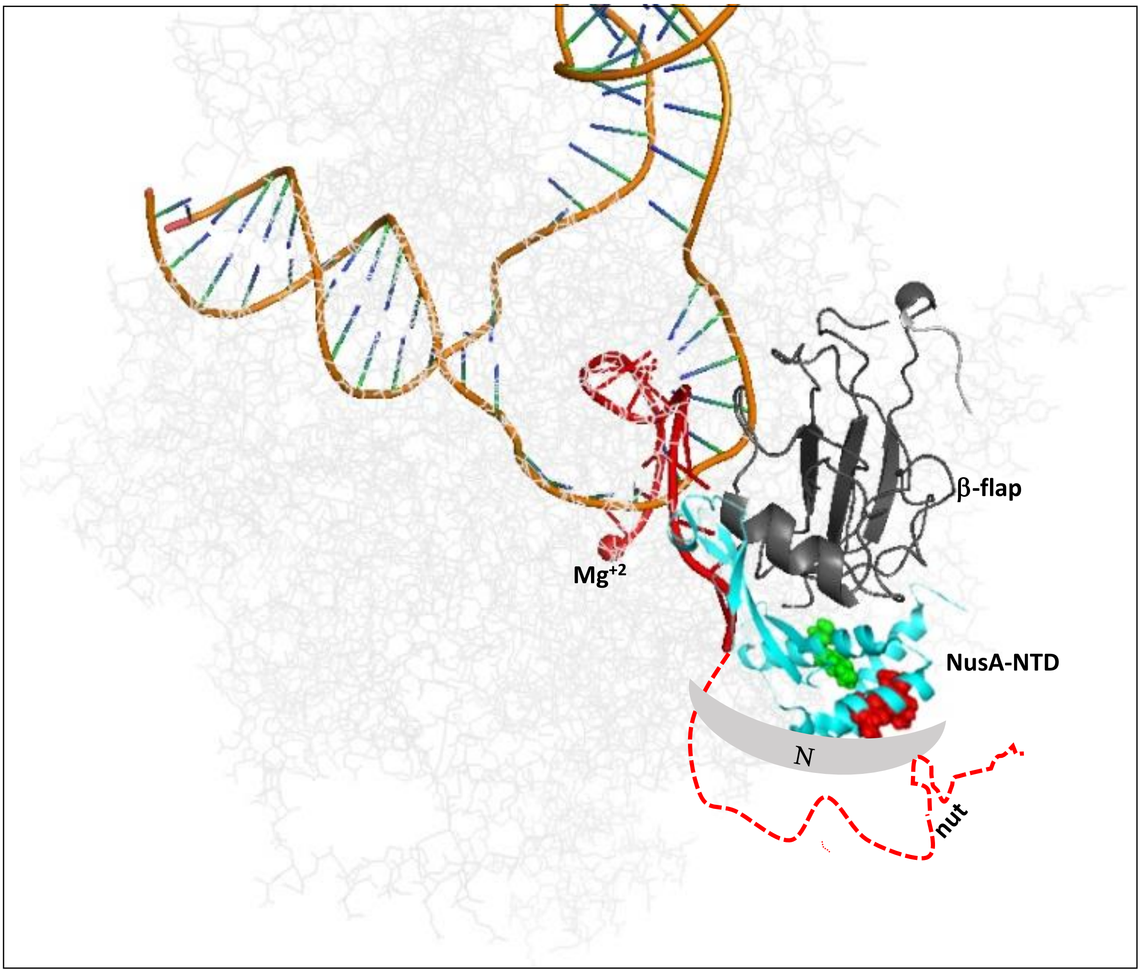

Figure S2

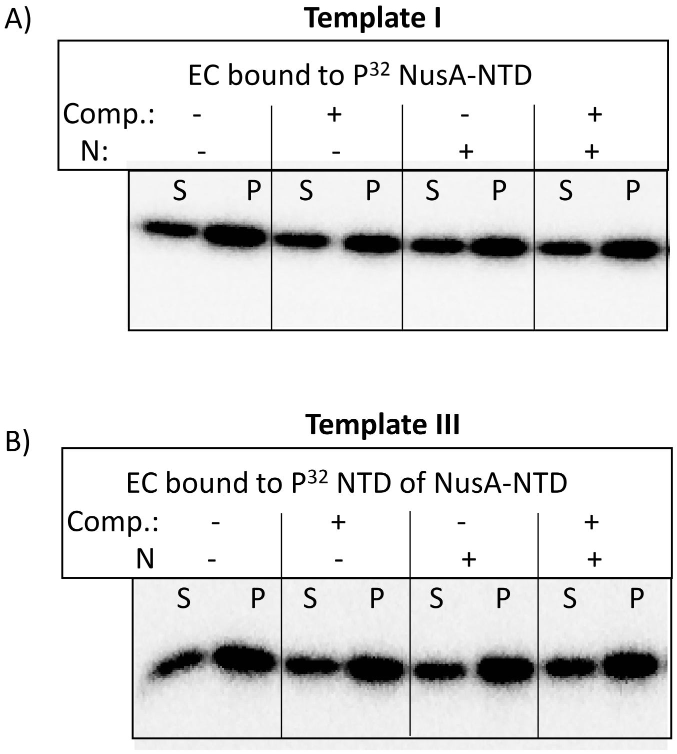
A)
Template I
EC bound to P32 NusA-NTD
Comp.: - + - +
N: - - + +
S P S P S P S P
B)
Template III
EC bound to P32 NTD of NusA-NTD
Comp.: - + - +
N - - + +
S P S P S P S P

Figure S3

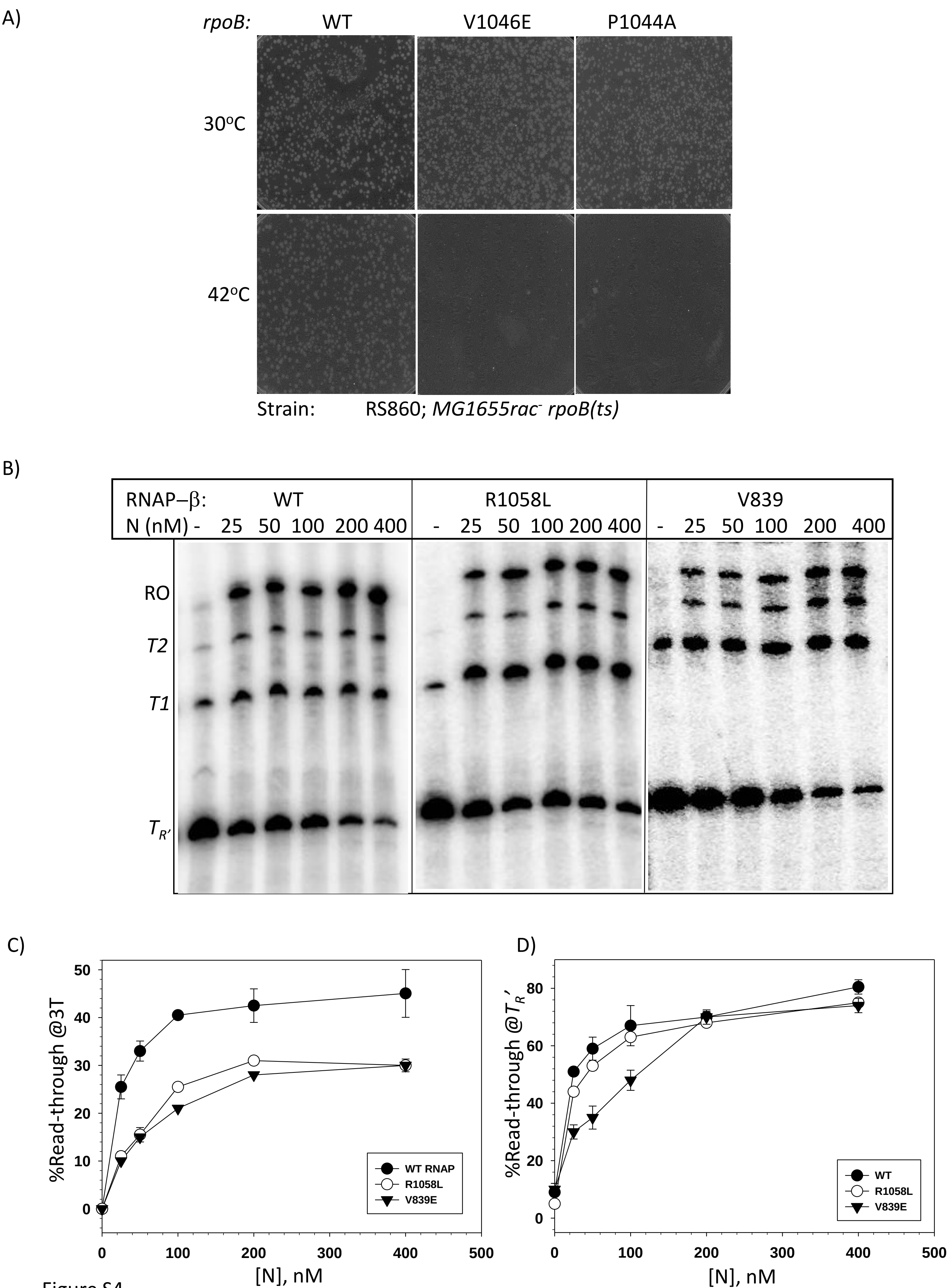
A)
rpoB: WT V1046E P1044A
30oC
42oC
Strain: RS860; MG1655rac- rpoB(ts)
B)
RNAP–β: WT R1058L V839
N (nM) - 25 50 100 200 400 - 25 50 100 200 400 - 25 50 100 200 400
RO
T2
T1
TR'
C)
%Read-through @3T
[N], nM
WT RNAP
R1058L
V839E
D)
%Read-through @TR'
[N], nM
WT
R1058L
V839E

Figure S4

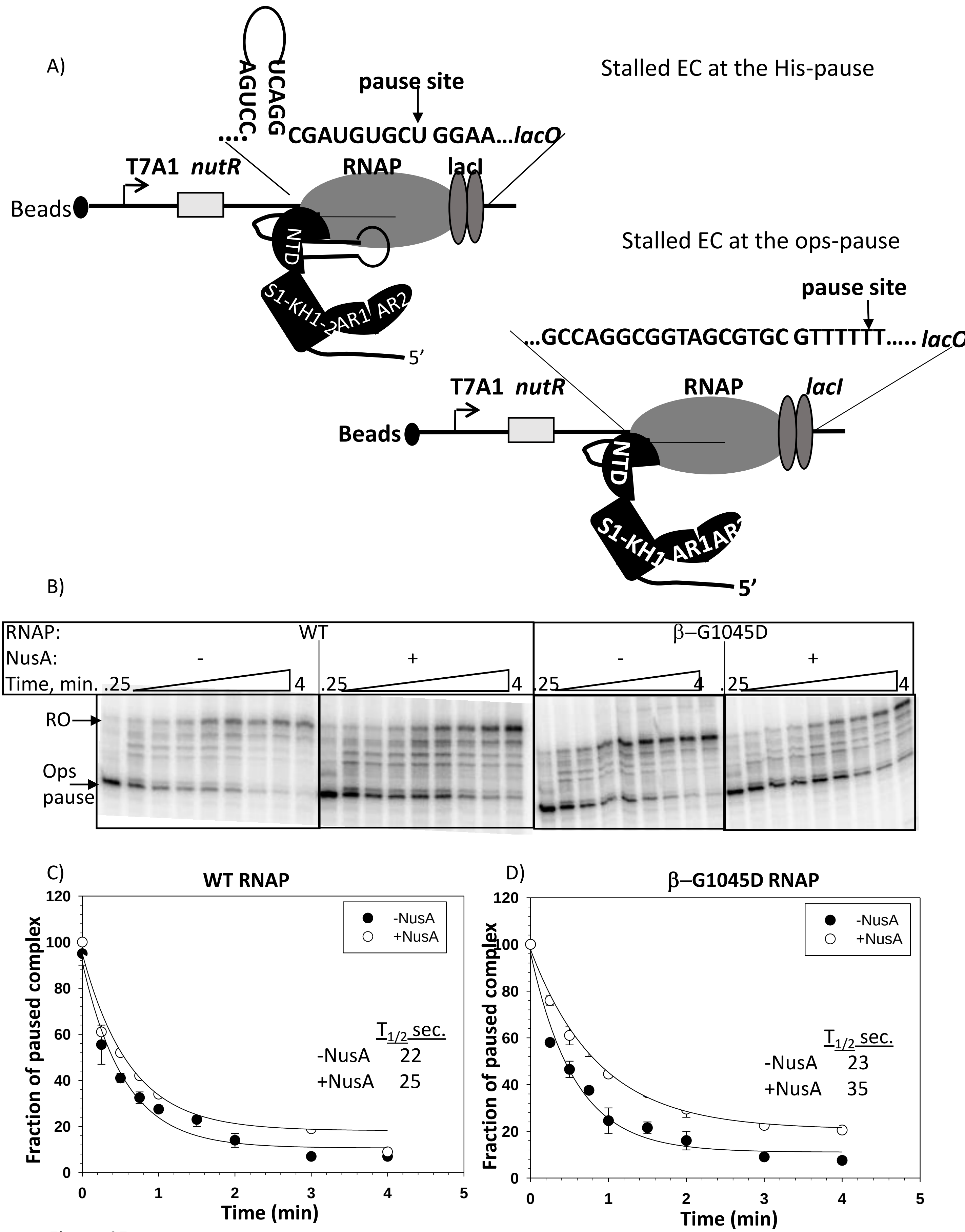
A)
Stalled EC at the His-pause
pause site
UCAGG
AGUCC
...:
CGAUGUGCU GGAA...lacO
T7A1 nutR
RNAP
lacI
Beads
NTD
S1-KH1-2
AR1
AR2
5'
Stalled EC at the ops-pause
pause site
...GCCAGGCGGTAGCGTGC GTTTTTT..... lacO
T7A1 nutR
RNAP
lacI
Beads
NTD
S1-KH1
AR1
5'
B)
RNAP:
WT
β–G1045D
NusA:
-
+
-
+
Time, min.
.25
4
RO
Ops pause
C)
WT RNAP
Fraction of paused complex
Time (min)
-NusA
+NusA
T1/2 sec.
-NusA 22
+NusA 25
D)
β–G1045D RNAP
Fraction of paused complex
Time (min)
-NusA
+NusA
T1/2 sec.
-NusA 23
+NusA 35

Figure S5

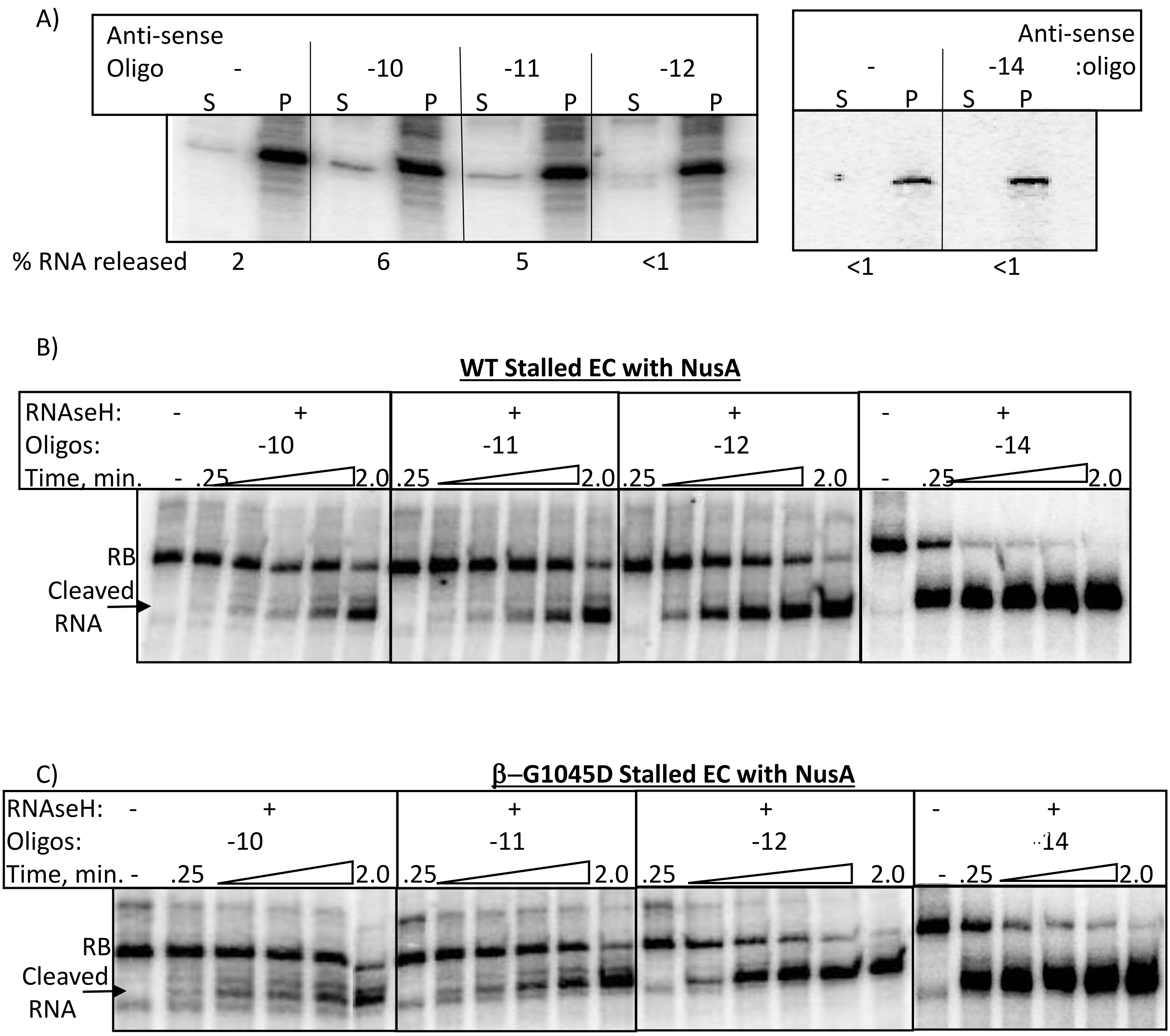
A)
Anti-sense
Oligo - -10 -11 -12
S P S P S P S P
% RNA released 2 6 5 <1
Anti-sense
- -14 :oligo
S P S P
<1 <1
B)
WT Stalled EC with NusA
RNAseH: - + + + - +
Oligos: -10 -11 -12 -14
Time, min. - .25 2.0 .25 2.0 .25 2.0 - .25 2.0
RB
Cleaved
RNA
C)
β–G1045D Stalled EC with NusA
RNAseH: - + + + - +
Oligos: -10 -11 -12 14
Time, min. - .25 2.0 .25 2.0 .25 2.0 - .25 2.0
RB
Cleaved
RNA

Figure S6

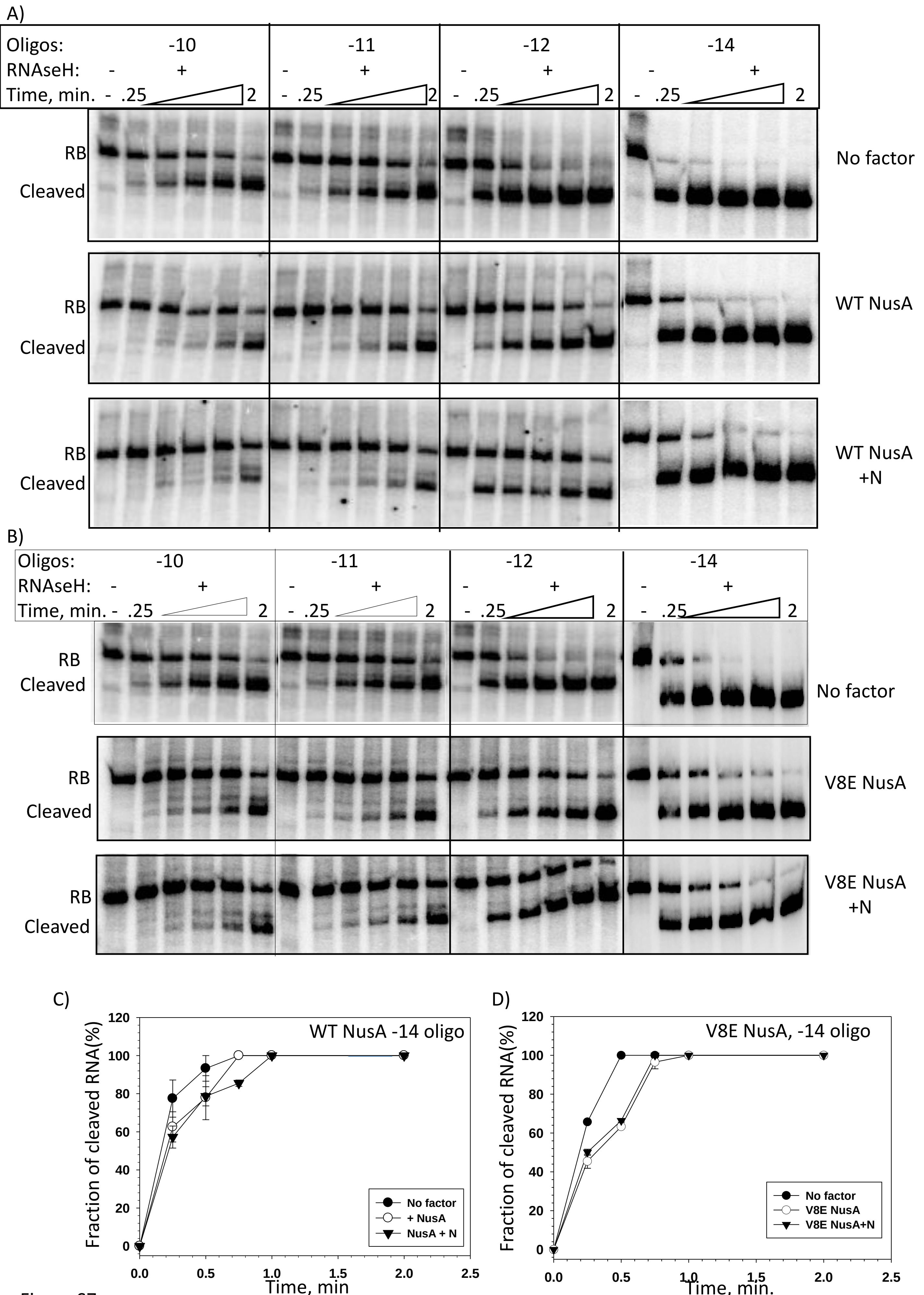
A)
Oligos: -10 -11 -12 -14
RNAseH: - + - + - + - +
Time, min. - .25 2 - .25 2 - .25 2 - .25 2
RB
Cleaved
No factor
WT NusA
WT NusA +N
B)
Oligos: -10 -11 -12 -14
RNAseH: - + - + - + - +
Time, min. - .25 2 - .25 2 - .25 2 - .25 2
RB
Cleaved
No factor
V8E NusA
V8E NusA +N
C)
WT NusA -14 oligo
Fraction of cleaved RNA(%)
Time, min
No factor
+ NusA
NusA + N
D)
V8E NusA, -14 oligo
Fraction of cleaved RNA(%)
Time, min.
No factor
V8E NusA
V8E NusA+N

Figure S7

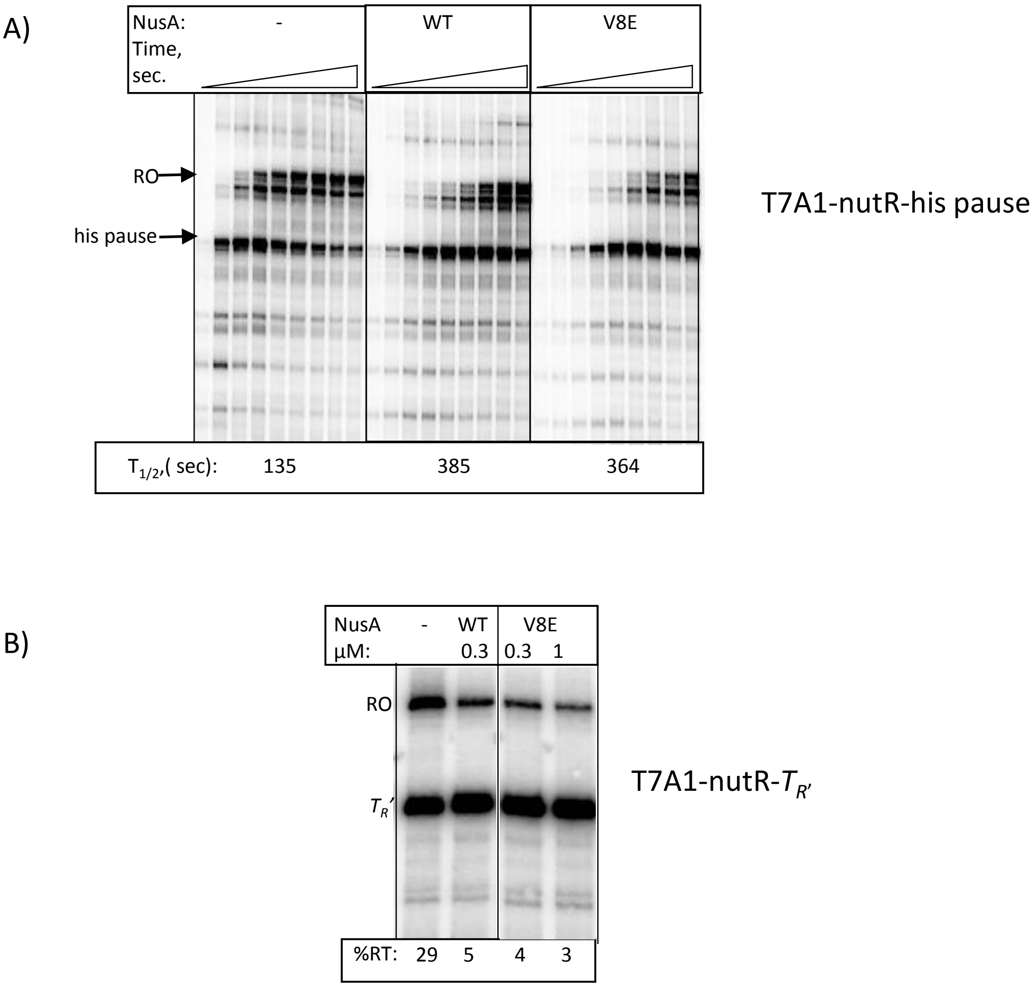

A)
NusA:
Time, sec.
-
WT
V8E
RO
his pause
T1/2,(sec): 135 385 364
T7A1-nutR-his pause
B)
NusA - WT V8E
µM: 0.3 0.3 1
RO
TR'
%RT: 29 5 4 3
T7A1-nutR-TR'

Figure S8

A)

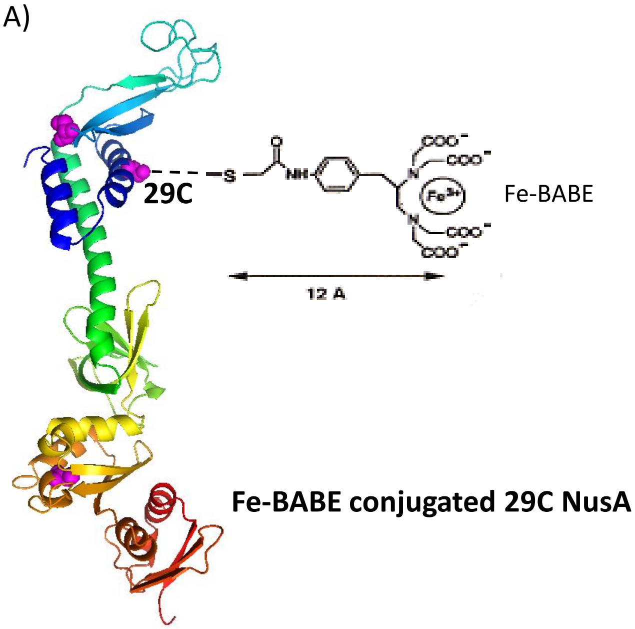

29C
S
NH
COO^-
N
Fe^{3+}
Fe-BABE
12 A
Fe-BABE conjugated 29C NusA

B)

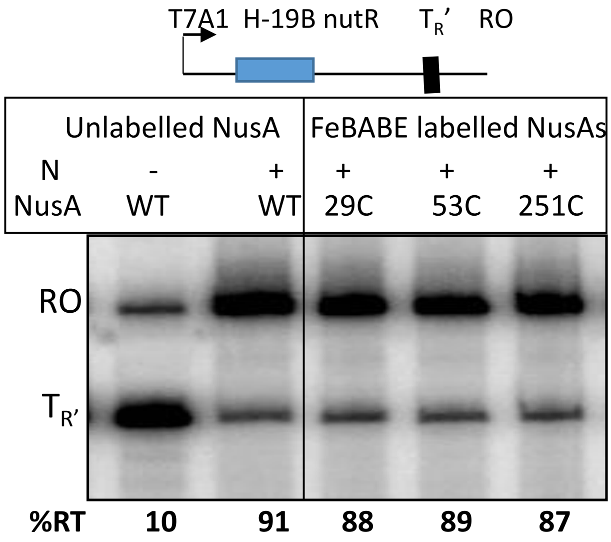

T7A1
H-19B nutR
T_R'
RO
Unlabelled NusA
FeBABE labelled NusAs
N
-
+
+
+
+
NusA
WT
WT
29C
53C
251C
RO
$T_{R'}$
%RT
10
91
88
89
87

Figure S9

A)

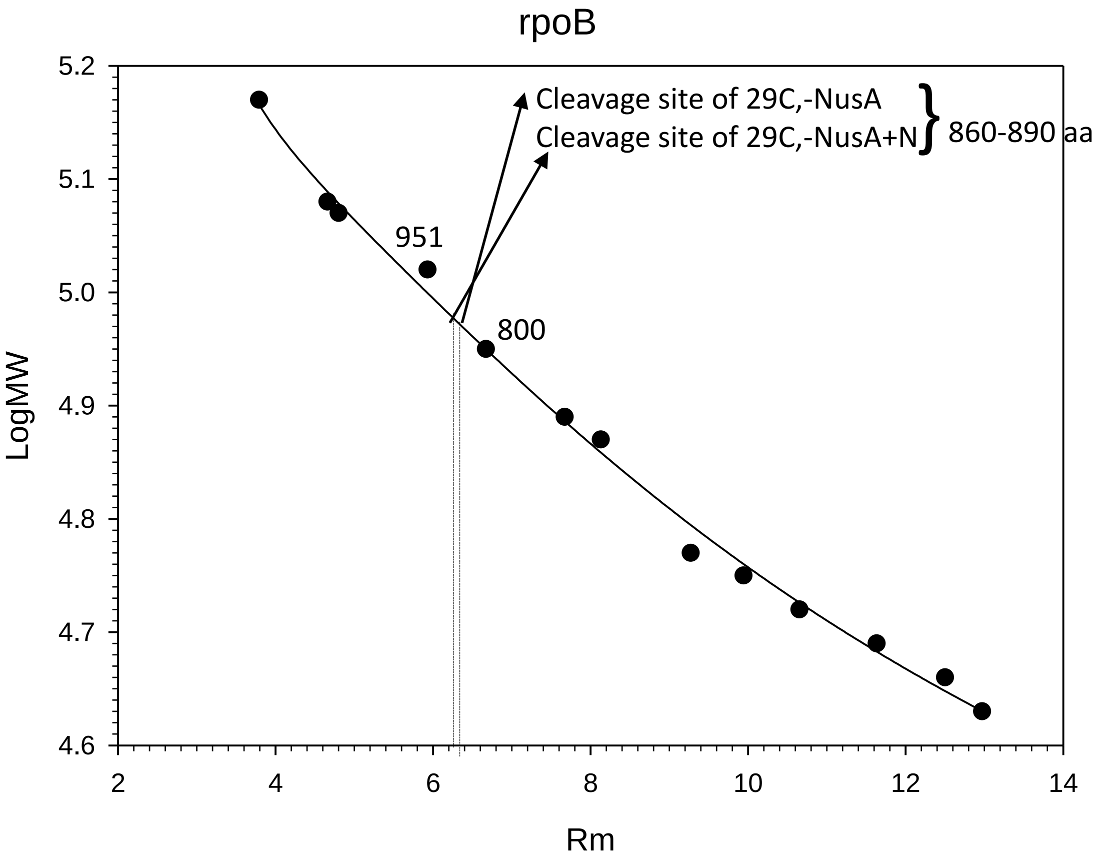

B)

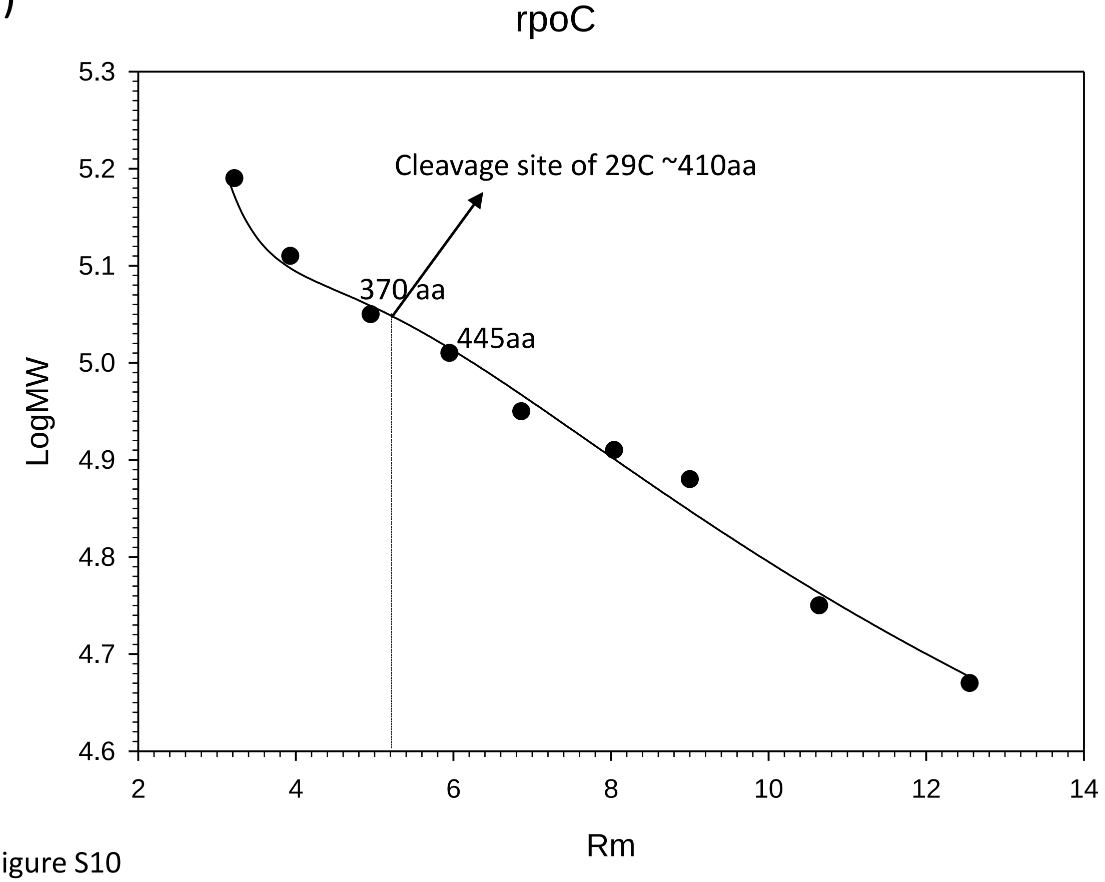

Figure S10

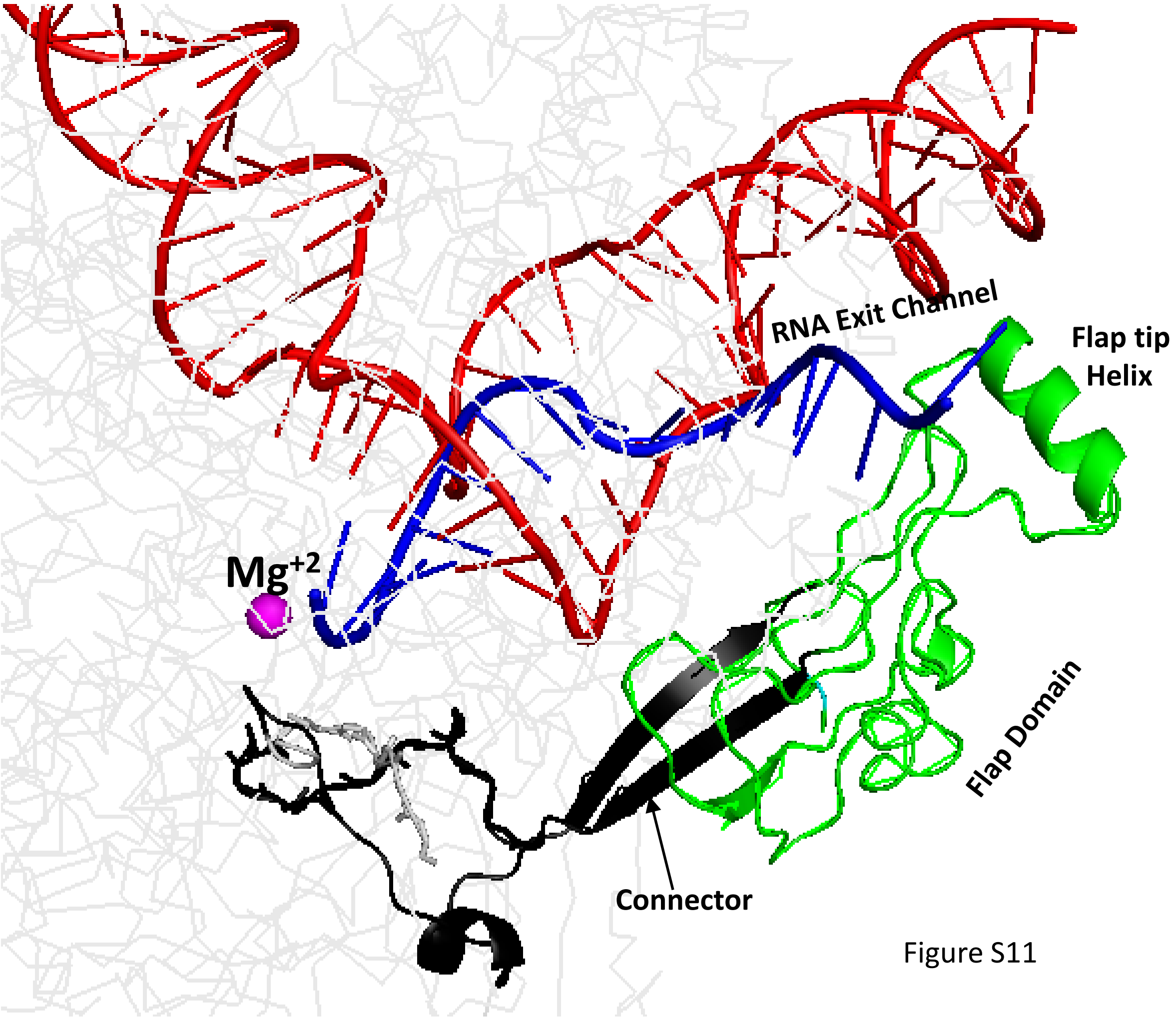

Figure S11
